# Algorithmic multiscale analysis for the FcRn mediated regulation of antibody PK in human

**DOI:** 10.1038/s41598-022-09846-x

**Published:** 2022-04-13

**Authors:** Dimitris G. Patsatzis, Shengjia Wu, Dhaval K. Shah, Dimitris A. Goussis

**Affiliations:** 1 School of Chemical Engineering, National Technical University of Athens, 15780 Athens, Greece; 2 Department of Pharmaceutical Sciences, School of Pharmacy and Pharmaceutical Sciences, The State University of New York at Buffalo, Buffalo, NY 14214-8033 USA; 3 Department of Mechanical Engineering, Khalifa University, 127788, Abu Dhabi, UAE

**Keywords:** Drug regulation, Pharmaceutics

## Abstract

A demonstration is provided on how algorithmic asymptotic analysis of multi-scale pharmacokinetics (PK) systems can provide (1) system level understanding and (2) predictions on the response of the model when parameters vary. Being algorithmic, this type of analysis is not hindered by the size or complexity of the model and requires no input from the investigator. The algorithm identifies the constraints that are generated by the fast part of the model and the components of the slow part of the model that drive the system within these constraints. The demonstration is based on a typical monoclonal antibody PK model. It is shown that the findings produced by the traditional methodologies, which require significant input by the investigator, can be produced algorithmically and more accurately. Moreover, additional insights are provided by the algorithm, which cannot be obtained by the traditional methodologies; notably, the dual influence of certain reactions depending on whether their fast or slow component dominates. The analysis reveals that the importance of physiological processes in determining the systemic exposure of monoclonal antibodies (mAb) varies with time. The analysis also confirms that the rate of mAb uptake by the cells, the binding affinity of mAb to neonatal Fc receptor (FcRn), and the intracellular degradation rate of mAb are the most sensitive parameters in determining systemic exposure of mAbs. The algorithmic framework for analysis introduced and the resulting novel insights can be used to engineer antibodies with desired PK properties.

## Introduction

Pharmacokinetics (PK) modeling is an indispensable tool in the drug development process, where mathematical models are implemented to characterize and predict the drug distribution and concentration over time profile^1^. Owning to the advance in computational and analytical methods, as well as the increasing need for preclinical-to-clinical translation and dosing optimization, the PK models are transforming from simple one and two compartmental models to more mechanistically based models. These models usually consist of a large number of complex equations and a much larger number of parameters, making it difficult to assess the impact of specific parameters, reactions and variables on a certain output^2–4^. The multiscale character of these models introduces another difficulty in assessing the influence of the various parameters and reactions, since the available in vivo data are usually collected in clinical trials with a frequency that adheres to the slow time scales^5^. As a result, processes that are characterized by fast time scales (such as the antibody-receptor binding) are not adequately resolved, so that identifiability problems arise^6^. Model reduction has been recognized as a viable remedy to the obstacles created by the complexity of the models of interest and their multiscale character. Several methodologies are available for model reduction, such as sensitivity analysis, singular perturbation (i.e., time-scale analysis), lumping, optimization etc; see Refs.^2,7–11^ for details.

The model reduction methods employed most frequently are the quasi-steady-state approximation (QSSA) and partial-equilibrium approximation (PEA); e.g.,^6,12–18^. Both methodologies are based on the multiscale character of the full model and are limiting cases of singular perturbation analysis^19–22^. The major drawback in their use is that their validity requires the identification of the number of fast time scales, the fast variables and fast reactions. Such identifications depend strongly on the value of the parameters employed and on the period of interest along a specific trajectory. These drawbacks are all manifested in the well known Michaelis-Menten (MM) model, for which the sQSSA, rQSSA and tQSSA reduced models were developed, each valid in distinct domains of the parameter space^23–25^. Depending on the parameters, the validity of these reduced models might be restricted in only a portion of a specific trajectory, so that more than one models must be employed if it is desired to approximate the full trajectory^26^. The drawbacks of QSSA and PEA and the fact that their use is not free of ambiguities are all well known. As a result, their implementation must always be subjected to verification^27–29^.

The traditional paper-and-pencil singular perturbation analysis (SPA) faces similar problems QSSA and PEA do; i.e., the number of fast variables and the proper non-dimensional form of the full model must be identified, both of which are strongly dependent on the value of the parameters employed or on the period of interest along a specific trajectory. However, a number of algorithms were developed that can produce most or all SPA does. All these algorithms are based on the geometric singular perturbation analysis (GSPA), which was developed in an effort to systematize the various SPA methodologies^30–32^. According to GSPA, the fast dynamics of a multiscale model are responsible for the development of low-dimensional surfaces in phase space, on which the process is constrained to evolve according to the slow dynamics^33^. The algorithm that provides all SPA does is the *Computational Singular Perturbation* (CSP), which identifies (1) the number of fast time scales and the reactions that are responsible for their development, (2) the variables that can be considered fast, (3) the constraints that develop due to fast dynamics and the reactions that participate in the related equilibria and (4) the reactions that drive the process within the constraints^34–36^. CSP is used extensively in combustion and chemical kinetic studies and recently in problems in biology and pharmacokinetics^26,37–43^. Regarding the subject of the present investigation, CSP has been already employed for the analysis of two target-mediated drug disposition (TMDD) models; a one-compartment model and a two-compartment with first order absorption one^40,41,43^. Among others, it was shown that algorithmic CSP tools provide (1) system-level understanding and (2) all necessary information to predict the response of the system when a change in the parameters is introduced.

Here, a simple antibody model, considering *Neonatal Fc Receptor* (FcRn) recycling of *Immunoglobulin G* (IgG), will be analyzed with CSP in order to demonstrate how system-level understanding can be obtained algorithmically. IgG typically shows an extended half-life compared to other protein molecules of similar size (i.e., 150 kDa) due to neonatal Fc receptor (FcRn) mediated recycling^44,45^. The binding between FcRn and IgG is negligible at physiological pH but strong at the acidic pH within the endosomes. This pH-dependent binding protects IgG from degradation in the lysosomal space, while allowing to release it back into the system circulation upon return to the cell surface^46^. Modification of the affinity between FcRn and IgG can alter antibody serum half-life as desired^47–49^. Based on the importance of FcRn binding on IgG half-life, mechanistic-based PK models of IgG usually incorporates FcRn binding component. The IgG enters the endosomal space via pinocytosis, and the binding of FcRn in endosomal space can salvage IgG from degradation. While this process has been described in the past, a systemic analysis of each parameters involved in this process has not been performed before. Consequently, here we have employed the CSP algorithm to gain more insight into the FcRn mediated recycling process of antibodies. The present investigation demonstrates the ability of CSP to provide insight that cannot be provided by the conventional methods.

The structure of the manuscript is as follows. First, the antibody model will be introduced and two QSSA-based reduced models will be generated, which are the appropriated ones for the parameter set employed here and are frequently encountered in the related literature^47,48,50,51^. A methodology for improving the accuracy of these two QSSA models will be presented and the physical understanding provided by the QSSA and the more accurate models will be compared. The CSP formalism will be briefly stated and the CSP analysis will follow. It will be demonstrated how CSP diagnostics can (1) provide system-level understanding and (2) suggest ways to modify the parameters of the model according to a desired response of the system.

## Model

**Figure 1:**
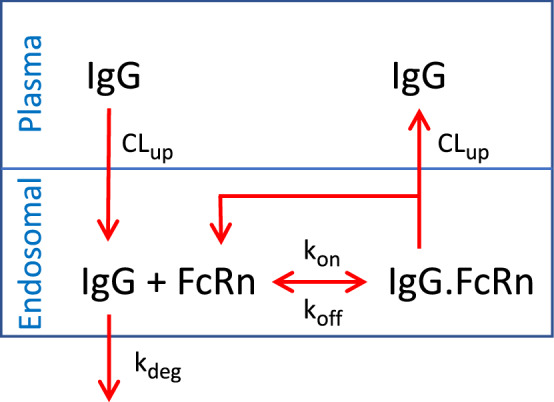
Schematic representation of the model formulating the interaction of IgG with the FcRn receptor, $R^1=k_1C^{IgG}_p$, $R^2=k_{deg}C^{IgG}_e$, $R^3=R^{3f}-R^{3b}=k_{on}C^{IgG}_e.C^{FcRn}_e-k_{off}C^{IgG.FcRn}_e$, $R^4=k_{1}C^{IgG.FcRn}_e$; $k_1={CL_{up}}/{V_e}$.

A schematic representation of the model employed here is depicted in Fig. 1. This model consists of two compartments: a plasma compartment and endosomal compartment. In the model, IgG can move from plasma compartment to endosomal compartment via pinocytosis (CL$_{up}$). IgG can then interact with FcRn via association ($k_{on}$) and dissociation ($k_{off}$) rate constants. Bound IgG will recycle back to plasma space (CL$_{up}$) and unbound IgG in the endosomal space will be eliminated with a elimination rate constant ($k_{deg}$). The model was able to capture the wildtype and Fc-mutated IgG PK profile in human after IV administration; e.g.,^46^. The process is governed by the system:

1 $$\begin{aligned}&\dfrac{d}{dt} \left[ \begin{array}{c} C^{IgG}_p  \\ C^{IgG}_e \\ C^{FcRn}_e \\ C^{IgG.FcRn}_e \end{array} \right] = \left[ \begin{array}{c} -a \\ +1 \\ 0 \\ 0 \end{array} \right] R^1+\left[ \begin{array}{c} 0 \\ -1 \\ 0 \\ 0 \end{array} \right] R^2+\left[ \begin{array}{c} 0 \\ -1 \\ -1 \\ +1 \end{array} \right] R^{3f}+\left[ \begin{array}{c} 0 \\ +1 \\ +1 \\ -1 \end{array} \right] R^{3b}+\left[ \begin{array}{c} a \\ 0 \\ +1 \\ -1 \end{array} \right] R^4 \end{aligned}$$

2 $$\begin{aligned}&C^{IgG}_p(0)=\frac{Dose}{V_p} \qquad C^{IgG}_e(0)=0 \qquad C^{FcRn}_e(0)=FcRn_o \qquad C^{IgG.FcRn}_e(0)=0 \end{aligned}$$

where

3 $$\begin{aligned}&R^1=k_1C^{IgG}_p \quad \ \ R^2=k_{deg}C^{IgG}_e \quad \ \ R^3=R^{3f}-R^{3b}=k_{on}C^{IgG}_e.C^{FcRn}_e-k_{off}C^{IgG.FcRn}_e \quad R^4=k_{1}C^{IgG.FcRn}_e \end{aligned}$$

and $a={V_e}/{V_p}$ and $k_1={CL_{up}}/{V_e}$. $C^{IgG}_p$ and $C^{IgG}_e$ denote IgG concentration in the plasma and the endosom, respectively, $C^{FcRn}_e$ denotes the FcRn concentration in the endosom and $C^{IgG.FcRn}_e$ denotes the IgG.FcRn concentration in the endosom. From the last two relations of Eq. (1), it follows that the conservation law $C^{FcRn}_e+C^{IgG.FcRn}_e=FcRn_o$ holds. The system in Eq. (1) can be cast in the compact form:

4 $$\begin{aligned} \dfrac{d\mathbf {y}}{dt} =\mathbf {S}_1R^1+\mathbf {S}_2R^2+\mathbf {S}_{3f}R^{3f}+\mathbf {S}_{3b}R^{3b}+\mathbf {S}_4R^4 = \mathbf {g}(\mathbf {y}) \end{aligned}$$

where $\mathbf {S}_i$ and $R^i$ denote the stoichiometric vector and rate of the i-th reaction, respectively.

## Conventional reduced models

The conventional methodology that is frequently employed for the construction of reduced models will be employed next. In particular, the QSSA for $C^{IgG}_e$ and $C^{FcRn}_e$ will be considered, which have previously employed in similar investigations; e.g.,^47,48,50,51^. These assumptions are the ones appropriate for the parameter set that was employed for the numerical demonstrations that follow. Of course, different assumptions might be appropriate if different parameter sets are considered.

### Reduced models based on the QSSA of $C^{IgG}_e$ and $C^{IgG.FcRn}_e$

Suppose that reaction 3f: $IgG+FcRn\rightarrow IgG.FcRn$ is very fast and that its reactant *IgG* is the fast variable, so that the following QSSA for $C^{IgG}_e$ holds:

5 $$\begin{aligned} R^{3f}-R^{3b} \approx R^1-R^2 \qquad \Rightarrow \qquad k_{on}C^{IgG}_e.C^{FcRn}_e-k_{off}C^{IgG.FcRn}_e\approx k_1C^{IgG}_p-k_{deg}C^{IgG}_e \end{aligned}$$

Substituting in Eq. (1), by eliminating the rate of the fast reaction 3f, yields:

6 $$\begin{aligned} \frac{dC^{IgG}_p}{dt}\approx & {} -a(R^1-R^4) \end{aligned}$$

7 $$\begin{aligned} \frac{dC^{IgG}_e}{dt}\approx \,& {} 0 \end{aligned}$$

8 $$\begin{aligned} \frac{dC^{FcRn}_e}{dt}\approx \,& {} -R^1+R^2+R^4 \ \end{aligned}$$

9 $$\begin{aligned} \frac{dC^{IgG.FcRn}_e}{dt}\approx \,& {} R^1-R^2-R^4 \end{aligned}$$

A reduced model can be constructed, using the algebraic relation in Eq. (5) and the differential Eqs. (6, 8, 9). Note that Eqs. (8, 9) keep satisfying the conservation law $C^{FcRn}_e+C^{IgG.FcRn}_e=FcRn_o$, which can replace either Eq. (8) or (9).

Suppose now that the 4th reaction $IgG.FcRn \rightarrow FcRn +a IgG$ is very fast and that its reactant *IgG*.*FcRn* is the fast variable, so that the following QSSA for $C^{IgG.FcRn}_e$ holds:

10 $$\begin{aligned} R^4 \approx R^1-R^2 \qquad \Rightarrow \qquad k_{1}C^{IgG.FcRn}_e \approx k_1C^{IgG}_p-k_{deg}C^{IgG}_e \end{aligned}$$

Substituting in Eqs. (6–9), by eliminating the rate of the fast reaction 4, yields:

11 $$\begin{aligned} \frac{dC^{IgG}_p}{dt}\approx & {} -aR^2 \end{aligned}$$

12 $$\begin{aligned} \frac{dC^{IgG}_e}{dt}\approx &\, {} 0 \end{aligned}$$

13 $$\begin{aligned} \frac{dC^{FcRn}_e}{dt}\approx & \,{} 0 \ \end{aligned}$$

14 $$\begin{aligned} \frac{dC^{IgG.FcRn}_e}{dt}\approx &\, {} 0 \end{aligned}$$

A new reduced model can be constructed, using the algebraic relations in Eqs. (5) and (10), the differential Eq. (11) and the conservation law $C^{FcRn}_e+C^{IgG.FcRn}_e=FcRn_o$.

### Higher order correction to the reduced models based on the QSSA of $C^{IgG}_e$ and $C^{IgG.FcRn}_e$

A higher order correction for the QSSA of $C^{IgG}_e$ is obtained by differentiating Eq. (5) with time^52,53^, substituting from Eq. (1) and solving for the rate of the fast reaction $R^{3f}$:

15 $$\begin{aligned} R^{3f}-R^{3b}\approx \dfrac{(1+r_1)R^1-R^2+(r_2-r_1)R^4}{1+r_2} \end{aligned}$$

or

16 $$\begin{aligned} k_{on}C^{IgG}_e.C^{FcRn}_e-k_{off}C^{IgG.FcRn}_e\approx \dfrac{(1+r_1)k_1C^{IgG}_p -k_{deg}C^{IgG}_e+(r_2-r_1)k_{1}C^{IgG.FcRn}_e}{1+r_2} \end{aligned}$$

where $r_1=k_1a/(k_{on}C^{FcRn}_e+k_{deg})$ and $r_2=(k_{on}C^{IgG}_e+k_{off})/(k_{on}C^{FcRn}_e+k_{deg})$. Substituting in Eq. (1) yields:

17 $$\begin{aligned} \frac{dC^{IgG}_p}{dt}\approx & {} -a(R^1-R^4) \end{aligned}$$

18 $$\begin{aligned} \frac{dC^{IgG}_e}{dt}\approx & {} \dfrac{\left( r_2-r_1\right) (R^1-R^4)-r_2R^2}{1+r_2} \end{aligned}$$

19 $$\begin{aligned} \frac{dC^{FcRn}_e}{dt}\approx & {} -\dfrac{\left( 1+r_1\right) (R^1-R^4)-R^2}{1+r_2} \end{aligned}$$

20 $$\begin{aligned} \frac{dC^{IgG.FcRn}_e}{dt}\approx & {} \dfrac{\left( 1+r_1\right) (R^1-R^4)-R^2}{1+r_2} \end{aligned}$$

A reduced model can be constructed by using Eqs. (17–20) or by using the algebraic relation Eq. (15) (which must be solved for the fast variable $C^{IgG}_e$) and the differential Eqs. (17, 19, 20). Note that this reduced model simplifies to the one in Eqs. (6–9) in the limits $r_1\rightarrow 0$ and $r_2\rightarrow 0$.

Similarly, a higher order correction for the QSSA of $C^{IgG.FcRn}_e$ is obtained by differentiating Eq. (10) with time^52,53^, substituting from Eqs. (17–20) and solving for the rate of the fast reaction $R^4$:

21 $$\begin{aligned} R^4\approx R^1-\dfrac{(1+m r_2)}{1+a+r_1(1-m)+r_2(a+m)}R^2=R^1-\Lambda R^2 \end{aligned}$$

or

22 $$\begin{aligned} k_{1}C^{IgG.FcRn}_e\approx k_1C^{IgG}_p-\Lambda k_{deg}C^{IgG}_e \end{aligned}$$

where $m=k_{deg}/k_1$, and then substituting in Eqs. (17–20):

23 $$\begin{aligned} \frac{dC^{IgG}_p}{dt}\approx & {} -a \Lambda R^2 \end{aligned}$$

24 $$\begin{aligned} \frac{dC^{IgG}_e}{dt}\approx & {} -\dfrac{r_2-(r_2-r_1)\Lambda }{1+r_2}R^2 \end{aligned}$$

25 $$\begin{aligned} \frac{dC^{FcRn}_e}{dt}\approx & {} \dfrac{1-(1+r_1)\Lambda }{1+r_2}R^2 \end{aligned}$$

26 $$\begin{aligned} \frac{dC^{IgG.FcRn}_e}{dt}\approx & {} -\dfrac{1-(1+r_1)\Lambda }{1+r_2}R^2 \end{aligned}$$

A reduced model can now be constructed by using Eqs. (23–26) or by using the algebraic relations Eqs. (15) and (21) (which must be solved for the fast variables $C^{IgG}_e$ and $C^{IgG.FcRn}_e$) and the differential Eqs. (23, 25). Note that this reduced model simplifies to the one in Eqs. (11–14) in the limits $r_1\rightarrow 0$, $r_2\rightarrow 0$ and $\Lambda \rightarrow 0$.

The conservation law $C^{FcRn}_e+C^{IgG.FcRn}_e=FcRn_o$ is still valid and can replace either of Eqs. (19,20) and either of Eqs. (25,26).

### Discussion

At this point it must be emphasized that for the construction of the reduced models in Eqs. (5–9), (10–14), (15–20) and (21–26) the investigator must identify (1) the proper number of QSSAs and (2) the variables and reactions that are considered fast. These are identifications, which are usually based on the experience of the investigator and must be subject to an *a posteriori* verification.

A comparison of (1) the reduced model in Eqs. (5–9), generated with one QSSA, with the equivalent one in Eqs. (15–20) and of (2) the reduced model in Eqs. (10–14), generated with two QSSAs, with the equivalent one in Eqs. (21–26), reveals that the models in Eqs. (15–20) and (21–26) provide more informations than those in Eqs. (5–9) and (10–14), respectively. For example, the differential equation for $C^{IgG}_e$ in Eq. (7) does not indicate the way the various reactions influence the evolution of this concentration. In contrast, the equivalent Eq. (18), indicates that the evolution of $C^{IgG}_e$ is determined by the 1st, 2nd and 4th reactions. This influence of the 4th reaction is absent in the related to $C^{IgG}_e$ equation of the full model, Eq. (1). However, it is included in Eq. (18) because its influence is exercised via the equilubrium in Eq. (15). Similar comments apply for the equations governing the evolution of $C^{FcRn}_e$ and $C^{IgG.FcRn}_e$.

It will be shown next that the reduced models in Eqs. (15–20) and (21–26) are more accurate than those in Eqs. (5–9) and (10–14). Given a complex model, the analytical derivation of such more accurate reduced models requires significant input from the investigator and considerable mathematic manipulations. However, as it will be shown next, this type of reduced models can be constructed algorithmically with the CSP algorithm. In addition, it will be demonstrated that the CSP-generated models provide a full system-level understanding, in contrast to the models in Eqs. (15–20) and (21–26). Such an understanding, is most useful when considering changes in the model or when exploring different operating conditions for the function of the model.

## CSP formalism and diagnostics

In CSP context, the 4-dim. model in Eq. (4) is cast in the form:

27 $$\begin{aligned} \dfrac{d\mathbf {y}}{dt} = \mathbf {a}_1f^1+ \mathbf {a}_2f^2+ \mathbf {a}_3f^3+ \mathbf {a}_4f^4 \end{aligned}$$

where the state vector is defined as $\mathbf {y}=[C^{IgG}_p,C^{IgG}_e,C^{FcRn}_e,C^{IgG.FcRn}_e]^T$, the 4-dim. column vectors $\mathbf {a}_n$ are the CSP basis vectors and $f^n$ are the related amplitudes:

28 $$\begin{aligned} f^n=\mathbf {b}^n \cdot \mathbf {g}(\mathbf {y}) =c^n_1R^1+c^n_2R^2+c^n_{3f}R^{3f}+c^n_{3b}R^{3b}+c^n_{4}R^4 \end{aligned}$$

where the 4-dim. row vectors $\mathbf {b}^n$ are the CSP dual basis vectors ($\mathbf {b}^i \cdot \mathbf {a}_j = \delta ^i_j$), $c^n_i=\mathbf {b}^n\cdot \mathbf {S}_i$ and $\mathbf {S}_i$ and $R^i$ are the stoichiometric vector and rate of the i-th reaction introduced in Eq. (4). The term $\mathbf {a}_nf^n$ in Eq. (27) represents a CSP mode and is characterized by a single time scale, say $\tau _n$. The CSP modes $\mathbf {a}_nf^n$ are ordered in Eq. (27) according to the value of $\tau _n$; first the fastest mode (smallest $\tau _n$), etc. Due to the conservation law $C^{FcRn}_e+C^{IgG.FcRn}_e=FcRn_o$, $f^4=0$ and $\tau _4=\infty$.

### CSP generated reduced models

When the fastest mode becomes exhausted, Eq. (27) reduces to:

29 $$\begin{aligned} f^1 \approx 0 \qquad \qquad \dfrac{d\mathbf {y}}{dt} \approx \mathbf {a}_2f^2+ \mathbf {a}_3f^3 \end{aligned}$$

Similarly, when the two fastest modes become exhausted, Eq. (27) reduces to:

30 $$\begin{aligned} f^1 \approx 0 \qquad \qquad f^2 \approx 0 \qquad \qquad \dfrac{d\mathbf {y}}{dt} \approx \mathbf {a}_3f^3 \end{aligned}$$

In general, considering an N-dim. system, when *M* fast modes become exhausted, the reduced model is:

31 $$\begin{aligned} f^r \approx 0 \qquad r=1, \ldots , M \qquad \qquad \dfrac{d\mathbf {y}}{dt} \approx \mathbf {a}_{M+1}f^{M+1}+\cdots + \mathbf {a}_Nf^N \end{aligned}$$

The M relations $f^i\approx 0$ define the constraints (i.e., the low dimensional surface in phase space) on which the system evolves according to the reduced model that is expressed by the differential equation in Eq. (31).

### CSP diagnostic tools

CSP provides a number of algorithmic tools that allow system level understanding. A detailed description of the CSP tools is provided in the Supplement. In summary, the following tools are available:

(i) *Amplitude Participation Index* (*API*) Identifies the reactions that contribute significantly to the formation of each of the *M* constraints, expressed by the relations $f^r \approx 0$ ($r=1,\ldots ,M$) in Eq. (31). The related index is $P^r_k$, which measures the contribution of the *k*-th reaction to the cancellations occurring in the expression $f^r \approx 0$. $P^r_k$ is scaled so that $\sum _{k=1}^{K}|P_k^{r}|=1$, where *K* is the number of reactions in the model^35,37,54^.
(ii) *Importance Index* (*II*) Identifies the reactions contributing the most in driving the evolution of each variable according to the system of differential equations in Eq. (31). The related index is $I^n_k$, which measures the influence of the *k*-th reaction in driving the *n*-th variable, within the established constraints. $I^n_k$ is scaled so that $\sum _{k=1}^K |I^n_k|=1$^35,37^. Positive (negative) $I^n_k$ indicates an influence of the *k*-th reaction in increasing (decreasing) the *n*-th variable.
(iii) *Time scale Participation Index* (*TPI*) Identifies the reactions that contribute significantly to the development of the time scale $\tau _n$ that characterises the n-th mode ($n=1,\ldots ,N$). The related index is $J^n_k$ measures the contribution of the *k*-th reaction to the *n*-th time scale $\tau _n$ and is scales so that $\sum _{k=1}^{K}|J_k^n|=1$^37,55,56^. A negative (positive) $J^n_k$ implies that the *k*-th reaction contributes to a dissipative (explosive) character of the *n*-th timescale $\tau _n$. By definition, dissipative (explosive) timescales relate to the components of the system that tend to drive it towards (away from) equilibrium^34,35^.
(iv) *Pointer* (*Po*) Identifies the variables related the most to the generation of the n-th mode. The related index is $D^n_i$ and is scaled so that $\sum _{n=1}^{N}D^n_i=1$^35,54,57^. A relatively large value of $D^n_i$ indicates that the *i*-th species is strongly associated to *n*-th CSP mode and the *n*-th timescale. A value of $D^n_i$ close to unity suggests the validity of the *Quasi Steady-State Approximation* (QSSA) for the *i*-th variable, while only two large values of $D^n_i$ indicate the validity of the *Partial Equilibrium Approximation* (PEA)^21^. The variables that are pointed by the *Pointer* of the *M* exhausted modes are those that are “slaved” to the remaining variables via the equilibrium relations $f^r \approx 0$ ($r=1 \dots M$) in Eq. (31) and are those that adjust the most when the related equilibrium relation is perturbed^54^.

Given a solution from a model in the form of Eq. (1), the CSP diagnostics (API, II, TPI and Po) can be computed algorithmically on the basis of the expressions provided in the Supplement, with relatively simple codes in Fortran, Python, Mathematica, MatLab, etc. The Fortran code employed here is publicly available in GitHub; see *Additional Information*. The required input are (1) the number of variables *N* and of unidirectional reactions *K*, (2) the stoichiometric vectors $\mathbf {S}_k$, the reaction rates $R^k$ and their gradient $\partial R^k / \partial y$.

## CSP results

The model parameters are presented in Table 1. Physiological values of human plasma and endosomal volume are used for the two compartments separately. The association ($k_{on}$) and dissociation ($k_{off}$) rate constant between IgG and FcRn and the IgG endosomal degradation rate ($k_{deg}$) were obtained from the literature^49,58^. The pinocytosis and exocytosis rate ($CL_{up}$) was calculated based on the reported per-unit endosomal uptake rate multiplying the endosomal volume.

**Table 1:** The parameter set employed for modelling the system in Eq. (1).

$V_p=3.10\ L$	$k_{on}=0.559\ (nM\cdot h)^{-1}$	$k_{off}=23.9\ h^{-1}$	$k_{deg}=25.0\ h^{-1}$
$V_e=0.34\ L$	$CL_{up}=0.167\ L \cdot h^{-1}$	$FcRn_0=4.98\, \times \, 10^4\ nM$	$Dose=24.0\, \times \, 10^4\ nmol$

**Figure 2:**
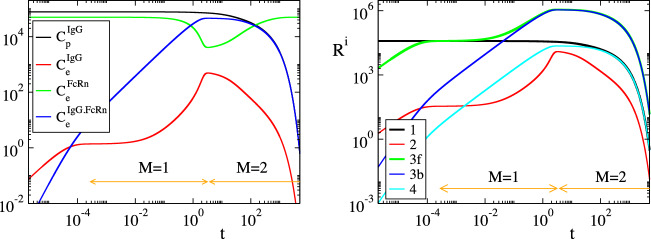
The evolution of the variables $C^{IgG}_p$, $C^{IgG}_e$, $C^{FcRn}_e$ and $C^{IgG.FcRn}_e$ (left) and of the rates $R^k$ (k = 1, 2, 3f, 3b, 4) (right). The periods indicated $M=1$ and $M=2$ refer to those in which one and two modes, respectively, are exhausted; i.e., in the $M=1$ period $f^1\approx 0$ (Eq. (29)) and in the $M=2$ period $f^1\approx 0$ and $f^2\approx 0$ (Eq. (30)).

**Figure 3:**
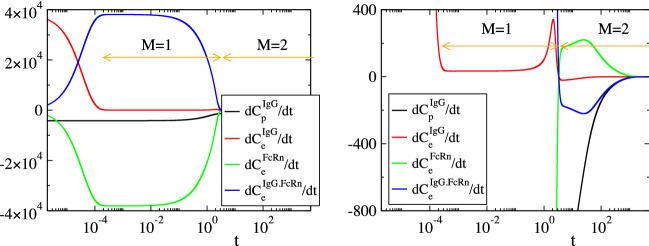
The evolution of the rate of change of $C^{IgG}_p$, $C^{IgG}_e$, $C^{FcRn}_e$ and $C^{IgG.FcRn}_e$.

**Figure 4:**
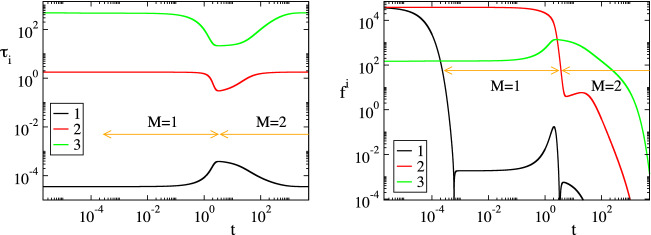
The evolution of the time scales $\tau _i$ (left) and of the amplitudes $f^i$ (right); $i=1,2,3$.

**Figure 5:**
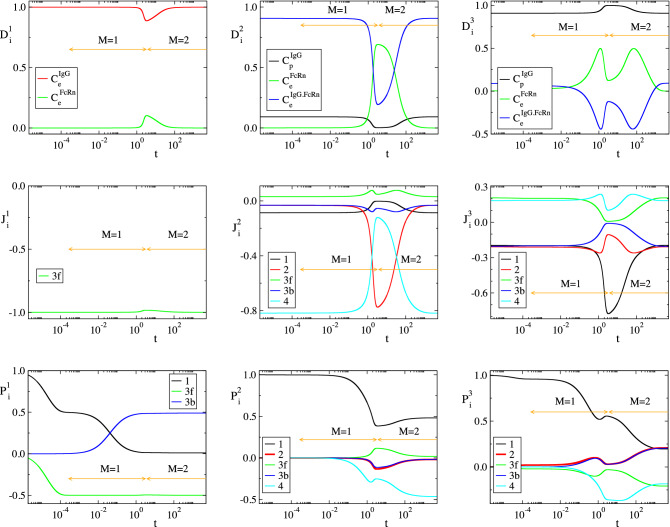
Evolution of CSP diagnostics for the three modes, $n=1, 2, 3$; TOP: Pointers $D^n_i$; $i=C^{IgG}_p$, $C^{IgG}_e$, $C^{FcRn}_e$, $C^{IgG.FcRn}_e$. MIDDLE: Time scale Participation Indices $J^n_k$; $k=1, 2, 3f, 3b, 4$. BOTTOM: Amplitude Participation Indices $P^n_k$; $k=1, 2, 3f, 3b, 4$.

Considering the parameters in Table 1, the solution of the model in Eq. (1) was analyzed with CSP and the results are displayed in Figs. 2, 3, 4 and 5. Figures 2 and 3 show that the profiles of (1) the concentrations, (2) the reaction rates $R^k$ and (3) the concentrations rate of change, exhibit periods of rapid changes and periods of very small variations. It will be shown that the latter periods are indicative of established equilibria.

Figure 4 displays the evolution of the timescales $\tau _n$ and of the amplitudes $f^n$ of the first three modes in Eq. (27) ($n=1,\dots ,3$); the forth mode $\mathbf {a}_4f^4$ is neglected, because it represents the conservation law $C^{FcRn}_e+C^{IgG.FcRn}_e=FcRn_o$, for which $\tau _4\equiv \infty$ and $f^4\equiv 0$. It is shown that significant gaps exits between $\tau _1$ and $\tau _2$ and between $\tau _2$ and $\tau _3$, so this feature established the multiscale character of the model. At each point in time the characteristic time scale is the fastest of those having a non-negligible amplitude; i.e., in the period $0<t<3\, \times \,10^{-4}\mathrm{h}$ the characteristic time scale is $\tau _1$ (M = 0 in Eq. (31)) and no reduced model is valid, in the period $3\, \times \,10^{-4} h<t<3 \mathrm{h}$ the characteristic time scale is $\tau _2$ (M=1) and the reduced model in Eq. (29) is valid and in the period $3 h<t$ the characteristic time scale is $\tau _3$ (M=2) and the reduced model in Eq. (30) is valid.

In Figs. 2, 3, 4 and 5 the periods in which a reduced model is valid is denoted by an arrow; $M=1$ indicates the period in which one mode is exhausted ($f^1\approx 0$) and a model of the form of Eq. (29) is valid, while $M=2$ indicates the period in which two modes are exhausted ($f^1\approx 0$ and $f^2\approx 0$) and a model of the form of Eq. (30) is valid.

### The M = 1 period

Consider the $M=1$ period, in which the first equilibrium $f^1\approx 0$ holds. According to Fig. 4, the fastest time scale $\tau _1$ that characterizes the development of this equilibrium there is much faster than $\tau _2$; about a four orders of magnitude difference. It is also shown that, right before the start of this period, the amplitude $f^1$ decreases considerably, as the first mode $\mathbf {a}_1f^1$ becomes exhausted, while the amplitude of the second mode $f^2$ remains at very large values. Figure 5 shows that the variable related to the first mode in the $M=1$ period is $C^{IgG}_e$ ($D^1_{C^{IgG}_e}\approx 1.0$) and that the reaction responsible for the emergence of $\tau _1$ is the IgG($C^{IgG}_e$)-consuming reaction 3f in the endosom $IgG+FcRn\rightarrow IgG.FcRn$ ($J^1_{3f}\approx -1.0$). Finally, it is shown that the equilibrium expressed by the relation $f^1\approx 0$ involves the equilibration of $R^{3f}$, first with $R^{1}$ ($R^{3f}\approx R^{1}$; $P^1_{3f}\approx -0.5$ and $P^1_{1}\approx 0.5$), then with $R^{1}+R^{3b}$ ($R^{3f}\approx R^{1}+R^{3b}$) and finally with $R^{3b}$ ($R^{3f}\approx R^{3b}$; $P^1_{3f}\approx -0.5$ and $P^1_{3b}\approx 0.5$). During the $M=1$ period, the dominant mode is the second one, the time scale $\tau _2$ of which is mainly generated by reaction 4 and by reaction 2 at the last part of the period, as shown in Fig. 5. Indicative values for all CSP diagnostics for this period ($M=1$) are provided in Table 2, as they were computed at $t=10^{-2}$.

**Table 2:** Solution data and CSP diagnostics at $t=10^{-2} \mathrm{h}$, where $M=1$.

$y_i$ ($\mathrm{nM}$)	$C^{IgG}_p$ = 77377.76	$C^{IgG}_e$ = 1.70	$C^{FcRn}_e$ = 49422.84	$C^{IgG.FcRn}_e$ = 377.16	
RHS ($\mathrm{nM h}^{-1}$)	$g^{IgG}_p$ = − 4148.09	$g^{IgG}_e$ = 33.84	$g^{FcRn}_e$ = − 37744.56	$g^{IgG.FcRn}_e$ = 37744.56	
$\tau _i$ ($\mathrm{h}^{-1}$)	$\tau _1$=0.36 10$^{-4}$	$\tau _2$=1.77	$\tau _3$=458.09		
$f^i$	f$^1$ = 1.86 10$^{-3}$	f$^2$ = 3.78 10$^{4}$	f$^1$ = 1.53 10$^2$		
n	IgG,p	IgG,e	FcRn,e	IgG.FcRn,e	
$D^1_n$	0.000	0.999	0.000	0.001	
$D^2_n$	0.091	0.000	0.001	0.906	
$D^3_n$	0.908	0.000	0.036	0.055	
k	1	2	3f	3b	4
$R^k$ ($\mathrm{nM h}^{-1}$)	38006.135	42.479	46943.920	9014.109	185.251
$J^1_k$	0.000	$-$ 0.001	$-$ 0.998	$-$ 0.001	0.000
$J^2_k$	$-$ 0.086	$-$ 0.034	0.032	$-$ 0.032	$-$ 0.817
$J^3_k$	$-$ 0.199	$-$ 0.211	0.203	$-$ 0.199	0.187
$c^1_k$	0.99910488	$-$ 0.99910293	$-$ 1.00000080	1.00000080	0.00089592
$c^2_k$	1.00209268	$-$ 0.90652448	0.00080178	$-$ 0.00080178	$-$ 1.00289446
$c^3_k$	0.00403610	0.09556626	$-$ 0.00008647	0.00008647	$-$ 0.00394963
$P^1_k$	0.404	$-$ 0.000	$-$ 0.500	0.096	0.000
$P^2_k$	0.992	$-$ 0.001	0.001	$-$ 0.000	$-$ 0.005
$P^3_k$	0.941	0.025	$-$ 0.025	0.005	$-$ 0.004
	$r_1$ = 1.94 10$^{-6}$	$r_2$ = 8.98 10$^{-4}$	a = 0.1096

**Table 3:** Relative error in approximating $(R^{3f}-R^{3b})$ at indicative points along the $M=1$ period: via the QSSA expression in Eq. (5) ($er^1_1$), the more accurate expression in Eq. (15) ($er^1_2$) and the CSP expression in Eq. (35) ($er^1_3$).

	t (h)	$10^{-2}$	$10^{-1}$	1	2	3	4
$R^{3f}-R^{3b}$	$er^1_1$	0.89$\, \times \,10^{-3}$	0.98$\, \times \,10^{-3}$	0.29$\, \times \,10^{-2}$	0.11$\, \times \,10^{-1}$	0.19$\, \times \,10^{-2}$	0.86$\, \times \,10^{-3}$
	$er^1_2$	0.39$\, \times \,10^{-5}$	0.42$\, \times \,10^{-5}$	0.98$\, \times \,10^{-5}$	0.33$\, \times \,10^{-4}$	0.58$\, \times \,10^{-4}$	0.62$\, \times \,10^{-4}$
	$er^1_3$	0.50$\, \times \,10^{-7}$	0.58$\, \times \,10^{-7}$	0.47$\, \times \,10^{-6}$	0.56$\, \times \,10^{-5}$	0.11$\, \times \,10^{-6}$	0.21$\, \times \,10^{-7}$

All these informations lead to the conclusion that in the $M=1$ period the QSSA of the $C^{IgG}_e$ variable is valid; i.e., $R^1-R^2-(R^{3f}-R^{3b})\approx 0$. In order to assess the accuracy of such an approximation, the following relative errors were computed:

32 $$\begin{aligned} er_1^1= & {} \dfrac{(R^{3f}-R^{3b})-(R^1-R^2)}{(R^{3f}-R^{3b})} \end{aligned}$$

33 $$\begin{aligned} er_2^1= & {} \dfrac{(R^{3f}-R^{3b})-\left( \dfrac{(1+r_1)R^1-R^2+(r_2-r_1)R^4}{1+r_2} \right) }{(R^{3f}-R^{3b})} \end{aligned}$$

34 $$\begin{aligned} er_3^1= & {} \dfrac{(R^{3f}-R^{3b})-\left( - \dfrac{ c^1_1R^1+c^1_2R^2+c^1_4R^4}{c^1_{3f}} \right) }{(R^{3f}-R^{3b})} \end{aligned}$$

where $er_1^1$ is the relative error in approximating $(R^{3f}-R^{3b})$ generated by the QSSA expression in Eq. (5), $er_2^1$ is the relative error generated by the higher order correction in Eq. (15) and $er_3^1$ is the relative error generated by the CSP derived expression:

35 $$\begin{aligned} f^1= c^1_1R^1+c^1_2R^2+c^1_{3f}R^{3f}+c^1_{3b}R^{3b}+c^1_4R^4 \approx 0 \end{aligned}$$

where, $c^1_{3f}=\mathbf {b}^{1}\cdot \mathbf {S}_{3f}=-\mathbf {b}^{1}\cdot \mathbf {S}_{3b}=-c^1_{3b}=c^1_3$. The results displayed in Table 3 indicate that the expression in Eq. (15) provided higher order accuracy to the standard QSSA in Eq. (5) ($er_2^1\ll er_1^1$) and the CSP-derived Eq. (35) provided higher to Eq. (15) accuracy ($er_3^1<er_2^1$). Note that using the $c^n_k$ values displayed in Table 2, the expression for the the first equilibrium, as derived by CSP in Eq. (35) at $t=10^{-2} \mathrm{h}$, yields:

36 $$\begin{aligned} (0.99910488)R^1-(0.99910293)R^2-(1.00000080)(R^{3f}-R^{3b})+(0.00089592)R^4 \approx 0 \end{aligned}$$

which is similar to the QSSA relation $R^1-R^2-(R^{3f}-R^{3b}) \approx 0$ in Eq. (5) and its extension in Eq. (15). The values of coefficients $c^n_k$ in this expression stay practically constant throughout the $M=1$ period. In addition, according to the $P^1_k$ values displayed in Fig. 5 , the major participants in the equilibrium across this period are $R^1$, $R^{3f}$ and $R^{3b}$; i.e., $R^2$ and $R^4$ provide negligible contributions and can be neglected. Therefore, Eq. (35) can be simplified as:

37 $$\begin{aligned} R^1 - (R^{3f}-R^{3b}) \approx 0 \end{aligned}$$

providing good accuracy across the $M=1$ period.

### The M = 2 period

Consider now the $M=2$ period, in which the two equilibria $f^1\approx 0$ and $f^2\approx 0$ hold. Regarding the first equilibrium, the CSP diagnostics are similar to those in the $M=1$ period; (1) the pointed variable $C^{IgG}_e$ is reactant of the fast reaction 3f, which is the main responsible for the emergence of $\tau _1$ and (2) the fact that $D^1_{C^{IgG}_e}\approx 1.0$ and that $R^{3f}\approx R^{3b}$ both indicate that the QSSA for $C^{IgG}_e$ is still valid, as in the $M=1$ period.

Regarding the second equilibrium Fig. 4 shows that the time scale $\tau _2$ that characterizes its development is much faster than $\tau _3$; about two orders of magnitude. It is also shown that the amplitude $f^2$ decreases considerably right before the start of the $M=2$ period, as the second mode $\mathbf {a}_2f^2$ becomes exhausted, while the amplitude of the third mode $f^3$ remains at very large values. Figure 5 shows that at the start of this period the variable related the most to this mode is $C^{FcRn}_e$, followed by $C^{IgG.FcRn}_e$, while for the remaining part of the period is only the latter variable ($D^2_{C^{IgG.FcRn}_e}\approx 0.9$). Figure 5 also shows that the reaction responsible for the emergence of $\tau _2$ is initially the IgG($C^{IgG}_e$)-consuming reaction 2 and then the IgG-FcRn($C^{IgG.FcRn}_e$)-consuming reaction 4. Finally, Fig. 5 shows that the equilibrium expressed by the relation $f^2\approx 0$ involves mainly the equilibration of $R^{4}$ and $R^{2}$ with $R^{1}$ ($R^{4}+R^{2}\approx R^{1}$), in which $R^{2}$ contributes only at the start of the $M=2$ period. Indicative values of all CSP diagnostics for this period are provided in Table 4, as they were computed at $t=10^{2}$.

All these informations lead to the conclusion that in the $M=2$ period the QSSA of the $C^{IgG}_e$ and $C^{IgG.FcRn}_e$ variables are valid. In order to assess the accuracy of these approximations, the relative errors $er_1^1$, $er_2^1$ and $er_3^1$ in approximating ($R^{3f}-R^{3b}$) in Eqs. (32–34) were computed along with the relative errors:

38 $$\begin{aligned} er_1^2= & {} \dfrac{R^4-(R^1-R^2)}{R^4} \end{aligned}$$

39 $$\begin{aligned} er_2^2= & {} \dfrac{R^4-\left( R^1-\dfrac{(1+m r_2)}{1+a+r_1(1-m)+r_2(a+m)}R^2 \right) }{R^4} \end{aligned}$$

40 $$\begin{aligned} er_3^2= & {} \dfrac{R^4-\left( - \dfrac{ c^2_1R^1+c^2_2R^2+c^2_{3f}R^{3f}+c^2_{3b}R^{3b}}{c^2_4} \right) }{R^4} \end{aligned}$$

where $er_1^2$ is the relative error in approximating $R^4$ generated by the QSSA expression in Eq. (10), $er_2^2$ is the relative error generated by the higher order correction in Eq. (21) and $er_3^2$ is the relative error generated by the CSP derived expression:

41 $$\begin{aligned} f^2= c^2_1R^1+c^2_2R^2+c^2_{3f}R^{3f}+c^2_{3b}R^{3b}+c^2_4R^4 \approx 0 \end{aligned}$$

where, $c^2_{3f}=\mathbf {b}^{2}\cdot \mathbf {S}_{3f}=-\mathbf {b}^{2}\cdot \mathbf {S}_{3b}=-c^2_{3b}=c^2_3$. The results displayed in Table 5 indicate that the expressions in Eqs. (15) and (21) provided higher order accuracy to the standard QSSA in Eq. (5) and (10) ($er_2^1\ll er_1^1$ and $er_2^2\ll er_1^2$) and the CSP-derived Eqs. (35) and (41) provided similar or better to Eqs. (15) and (21) accuracy ($er_3^1 \ll er_2^1$ and $er_3^2 \le er_2^2$).

**Table 4:** Solution data and CSP diagnostics at $t=10^2\, \mathrm{h}$, where $M=2$.

$y_i$ ($\mathrm{nM}$)	$C^{IgG}_p$ = 31346.03	$C^{IgG}_e$ = 58.84	$C^{FcRn}_e$ = 21202.58	$C^{IgG.FcRn}_e$ = 28597.42	
RHS ($\mathrm{nM h}^{-1}$)	$g^{IgG}_p$ = − 148.07	$g^{IgG}_e$ =− 0.58	$g^{FcRn}_e$ = 120.40	$g^{IgG.FcRn}_e$ = − 120.40	
$\tau _i$ ($h^{-1}$)	$\tau _1$=0.84 10$^{-4}$	$\tau _2$ = 1.53	$\tau _3$ = 100.82		
$f^i$	f$^1$ = 0.55 10$^{-6}$	f$^2$ = 0.37	f$^1$ = 148.03		
n	IgG,p	IgG,e	FcRn,e	IgG.FcRn,e	
$D^1_n$	0.000	0.995	0.003	0.002	
$D^2_n$	0.068	0.004	0.096	0.831	
$D^3_n$	0.932	0.000	0.476	− 0.407	
k	1	2	3f	3b	4
$R^k$ ($\mathrm{nM h}^{-1}$)	15369.432	1471.039	697404.378	683478.404	14046.381
$J^1_k$	0.000	− 0.002	− 0.996	0.000	− 0.002
$J^2_k$	− 0.060	− 0.149	0.060	− 0.061	− 0.668
$J^3_k$	− 0.290	− 0.258	0.109	− 0.107	0.234
$c^1_k$	0.99525512	− 0.99525062	− 1.00000977	1.00000977	0.00475466
$c^2_k$	1.00855828	− 0.92519772	0.00190065	− 0.00190065	− 1.01045892
$c^3_kk$	0.01881054	0.08335607	− 0.00017575	0.00017575	− 0.01863478
$P^1_k$	0.011	− 0.001	− 0.499	0.489	0.000
$P^2_k$	0.461	− 0.040	0.039	− 0.038	− 0.421
$P^3_k$	0.316	0.134	− 0.134	0.131	− 0.286
	$r_1$ = 4.53 10$^{-6}$	$r_2$ = 4.78 10$^{-3}$	a = 0.1096

**Table 5:** Relative error in approximating $(R^{3f}-R^{3b})$ (top) and $R^4$ (bottom) at indicative points along the $M=2$ period: TOP: via the QSSA expression in Eq. (5) ($er^1_1$), the more accurate expression in Eq. (15) ($er^1_2$) and the CSP expression in Eq. (35) ($er^1_3$), BOTTOM: via the QSSA expression in Eq. (10) ($er^2_1$), the more accurate expression in Eq. (21) ($er^2_2$) and the CSP expression in Eq. (41) ($er^2_3$).

	t (h)	5	10	20	50	100	1000
$R^{3f}-R^{3b}$	$er^1_1$	0.92$\, \times \,10^{-3}$	0.70$\, \times \,10^{-3}$	0.42$\, \times \,10^{-3}$	0.12$\, \times \,10^{-3}$	0.41$\, \times \,10^{-4}$	0.44$\, \times \,10^{-5}$
	$er^1_2$	0.59$\, \times \,10^{-4}$	0.47$\, \times \,10^{-4}$	0.32$\, \times \,10^{-4}$	0.16$\, \times \,10^{-4}$	0.99$\, \times \,10^{-5}$	0.42$\, \times \,10^{-5}$
	$er^1_3$	0.24$\, \times \,10^{-7}$	0.15$\, \times \,10^{-7}$	0.55$\, \times \,10^{-8}$	0.43$\, \times \,10^{-9}$	0.39$\, \times \,10^{-10}$	0.24$\, \times \,10^{-13}$
$R^4$	$er^2_1$	0.88$\, \times \,10^{-2}$	0.97$\, \times \,10^{-2}$	0.10$\, \times \,10^{-1}$	0.10$\, \times \,10^{-1}$	0.86$\, \times \,10^{-2}$	0.44$\, \times \,10^{-2}$
	$er^2_2$	0.87$\, \times \,10^{-4}$	0.48$\, \times \,10^{-4}$	0.13$\, \times \,10^{-4}$	0.12$\, \times \,10^{-3}$	0.10$\, \times \,10^{-3}$	0.18$\, \times \,10^{-4}$
	$er^2_3$	0.22$\, \times \,10^{-4}$	0.23$\, \times \,10^{-4}$	0.28$\, \times \,10^{-4}$	0.12$\, \times \,10^{-3}$	0.26$\, \times \,10^{-4}$	0.24$\, \times \,10^{-7}$

As Table 5 indicates, the accuracy in approximating $(R^{3f}-R^{3b})$ with the CSP-derived expression is higher than that in approximating $R^4$. This feature relates to the fact that the accuracy of the constraint $f^M\approx 0$, which is generated after the *M*-th time scale becomes exhausted (so that the characteristic time scale changes from $\tau _{M}$ to $\tau _{M+1}$), is proportional to the size of the ratio $\tau _M/\tau _{M+1}$; i.e., the smaller this ratio the higher the accuracy^59,60^. Accordingly, the accuracy of the CSP expression for approximating $(R^{3f}-R^{3b})$ in Eq. (35), which becomes valid after the fastest time scale $\tau _1$ becomes exhausted, is proportional to $\tau _1/\tau _2$, while the accuracy of the CSP expression for approximating $R^4$ in Eq. (41), which becomes valid after $\tau _2$ becomes exhausted, is proportional to $\tau _2/\tau _3$. Figure 4 shows that $\tau _1/\tau _2 \ll \tau _2/\tau _3$. Therefore, the accuracy of the former approximation will be higher than the latter, in accordance to the results in Table 5. Note that using the $c^n_k$ values displayed in Table 4, in the expressions for the first and second equilibria, as derived by CSP in Eqs. (35) and (41) at $t=10^2 \mathrm{h}$, yield:

42 $$\begin{aligned}&(0.99525512)R^1-(0.99525062)R^2-(1.00000977)(R^{3f}-R^{3b})+(0.00475466)R^4 \approx 0 \end{aligned}$$

43 $$\begin{aligned}&(1.00855828)R^1-(0.92519772)R^2+(0.00190065)(R^{3f}-R^{3b})-(1.01045892)R^4 \approx 0 \end{aligned}$$

As in the $M=1$ period discussed previously, Eq. (42) is still similar to the QSSA relation $R^1-R^2-(R^{3f}-R^{3b}) \approx 0$ in Eq. (5) and its extension in Eq. (15). Equation (43) is similar to the QSSA relation $R^1-R^2-R^{4} \approx 0$ in Eq. (10) and resembles to its extension in Eq. (21), in which $R^{3f}-R^{3b}$ is however not present. The values of coefficients $c^n_k$ in these two expressions vary negligibly in the M = 1 period. In addition, according to the $P^n_k$ values displayed in Fig. 5 (1) the major participants in the first equilibrium ($n=1$) across the $M=2$ period are $R^{3f}$ and $R^{3b}$, while $R^1$, $R^2$ and $R^4$ provide negligible contributions and (2) the major participants in the second equilibrium ($n=2$) are $R^1$ and $R^4$, while $R^2$, $R^{3f}$ and $R^{3b}$ provide small but no negligible contributions only at the early stage of this period. As a result, the previous expressions can be simplified throughout the $M=2$ period as:

44 $$\begin{aligned} R^{3f}-R^{3b} \approx 0 \qquad \qquad R^1 - R^4 +\left[ c_2^2R^2+c^2_3(R^{3f}-R^{3b}) \right] \approx 0 \end{aligned}$$

where the terms in the brackets provide smaller contributions.

### The reactions driving the process

**Figure 6:**
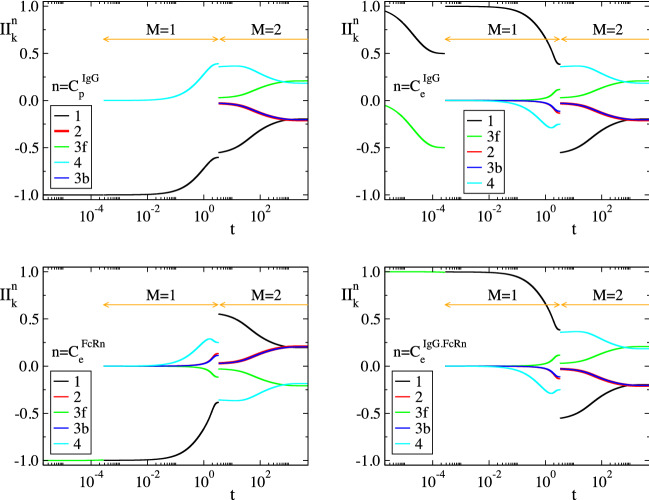
The participation index $II^n_k$ ($k=1,2,3f,3b,4$) for all four variables ($n=C_p^{IgG}$,$C_e^{IgG}$,$C_e^{FcRn}$,$C_e^{IgG.FcRn}$) in the periods where $M=0,1,2$.

When the *M* equilibria have been established, as it is expressed by the algebraic relations $f^r \approx 0$ ($r=1 \ldots M$), the system is driven by the dominant reactions in the simplified system of differential equations in Eq. (31). These reactions are identified by the *Importance Index*, introduced in “CSP diagnostic tools” Section and displayed in Fig. 6; $II^n_k>0$ ($II^n_k<0$) implies that the $n-th$ variable is produced (consumed) by the $k-th$ reaction.

Figure 6 shows that initially (while $M=0$), $C_p^{IgG}$ is consumed only by reaction 1 ($IgG_p \rightarrow IgG_e$), $C_e^{IgG}$ is produced by reaction 1 and then gradually consumed by reaction 3f ($IgG_e+FcRn_e \rightarrow IgG_e\cdot FcRn_e$) and $C_e^{FcRn}$ and $C_e^{IgG.FcRn}$ are gradually consumed and produced, respectively, by reaction 3f. These findings can also be predicted by inspection of the governing equations in Eq. (1).

After the first equilibrium is established ($M=1$: $R^1-R^2-(R^{3f}-R^{3b})\approx 0$) and the 1st mode becomes exhausted, the process is driven by the remaining 2nd and 3rd modes; see Eq. (29), which is valid in the $M=1$ period. Figure 6 shows that the action of the two active modes in this period refers mainly to reactions 1 ($IgG_p \rightarrow IgG_e$) and 4 ($IgG_e.FcRn \rightarrow IgG_p+FcRn$) and partly to reaction 2 ($IgG_e \rightarrow$), while the contributions of reactions 3f and 3b manifest towards the end of this period cancelling each other. In particular, Fig. 6 shows that $C_p^{IgG}$ is consumed by reaction 1 ($II^{IgG,p}_1<0$) and is produced by reaction 4 ($II^{IgG,p}_4>0$). $C_e^{IgG}$ and $C_e^{IgG.FcRn}$ are produced by reaction 1 ($II^{IgG,e}_1>0$, $II^{IgG.FcRn,e}_1>0$) and during the last stage of this period are consumed mainly by reaction 4 ($II^{IgG,e}_4<0$, $II^{IgG.FcRn,e}_4<0$) and partly by reaction 2 ($II^{IgG,e}_2<0$, $II^{IgG.FcRn,e}_2<0$). These reactions have exactly the opposite influence to the evolution of $C_e^{FcRn}$ (mainly $II^{FcRn,e}_1<0$, $II^{FcRn,e}_4>0$ and partly $II^{FcRn,e}_2>0$). At the end of this period, reactions 3f and 3b exhibit a minor influence to the evolution of the three variables in the endosom, but their contributions cancel each other.

When the second equilibrium is established, both the 1st and 2nd modes are exhausted ($M=1$: $R^{3f}-R^{3b}\approx 0 \text {and} R^1-R^2-R^4\approx 0$) and it is only the 3rd mode that drives the system; see Eq. (30), which is valid in the $M=2$ period. It is shown in Fig. 6 that in this period reactions 1 and 2 tend to decrease $C_p^{IgG}$, $C_e^{IgG}$ and $C_e^{IgG.FcRn}$ ($II^{IgG,p}_1<0$, $II^{IgG,p}_2<0$, $II^{IgG,e}_1<0$, $II^{IgG,e}_2<0$, $II^{IgG.FcRn,e}_1<0$, $II^{IgG.FcRn,e}_2<0$) and to increase $C_e^{FcRn}$ ($II^{FcRn,e}_1>0$, $II^{FcRn,e}_2>0$), reaction 4 has the opposite tendencies ($II^{IgG,p}_4>0$, $II^{IgG,e}_4>0$, $II^{IgG.FcRn,e}_4>0$, $II^{FcRn,e}_4<0$), while the contributions of reactions 3f and 3b cancel each other.

Some of the features displayed in Fig. 6 can be detected from the original model in Eq. (1) in the period where no mode is exhausted ($M=0$), the reduced models in Eqs. (6–9) or Eqs. (17–20) that are valid in the $M=1$ period and the reduced models in Eqs. (11–14) or (23–26) that are valid in the $M=2$ period; e.g., the influence of reactions 1, 3f and 4 during the initial and the $M=1$ periods or the transition from reaction 3f to reaction 1 as the major driving reaction for the evolution of $C_e^{FcRn}$ and $C_e^{IgG.FcRn}$ at the start of the $M=1$ period.

However, there are features that cannot be foreseen by inspection of the full or the reduced models; even in the case where these models are simple, as those considered here. For example, Fig. 6 shows that during the transition from the $M=1$ to the $M=2$ period, the influence of reactions 1 and 4 reverses. That is, in the $M=1$ period reaction 1 tends to increase $C_e^{IgG}$ and $C_e^{IgG.FcRn}$ and to decrease $C_e^{FcRn}$ ($II^{IgG,e}_1>0$, $II^{IgG.FcRn,e}_1>0$, $II^{FcRn,e}_1<0$), while in the $M=2$ period its influence reverses; i.e., reaction 1 tends to decrease $C_e^{IgG}$ and $C_e^{IgG.FcRn}$ and to increase $C_e^{FcRn}$ ($II^{IgG,e}_1<0$, $II^{IgG.FcRn,e}_1<0$, $II^{FcRn,e}_1>0$). Reaction 4 exhibits exactly the opposite behavior to reaction 1; i.e., tends to decrease $C_e^{IgG}$ and $C_e^{IgG.FcRn}$ and to increase $C_e^{FcRn}$ in the $M=1$ period and exhibits the opposite tendency in the $M=2$ period. Although the influence of these reactions in the $M=1$ period can be predicted from the relevant reduced model in Eqs. (17–20), their influence in the $M=2$ period cannot be predicted, since reactions 1 and 4 are not present in the appropriate reduced model in Eqs. (23–26).

Another feature that cannot be detected by inspecting the existing models relates to the influence of reactions 3f and 3b. Figure 6 shows that these reactions are shown to exercise a certain influence in the evolution of the system during the last stage of the $M=1$ period and throughout the $M=2$ one, in contrast to what is suggested by the reduced models derived in “Conventional reduced models” Section, in which these reactions are absent.

**Figure 7:**
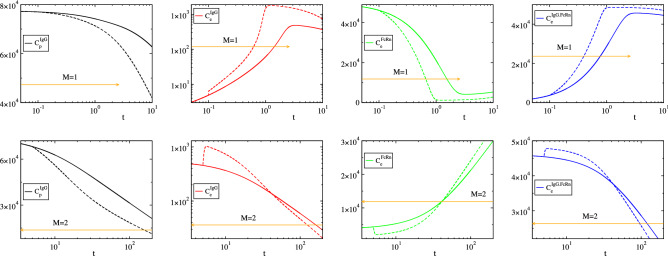
Concentration profiles for the reference case (parameter values in Table 1, solid curves) and for the case in which the reaction rate constant of $R^1$ is doubled (dashed curves). The perturbation is enforced from $t=0.1\, \mathrm{h}$ (top) and $t=5\, \mathrm{h}$ (bottom). The indicated $M=1$ and $M=2$ periods refer to the reference case.

**Figure 8:**
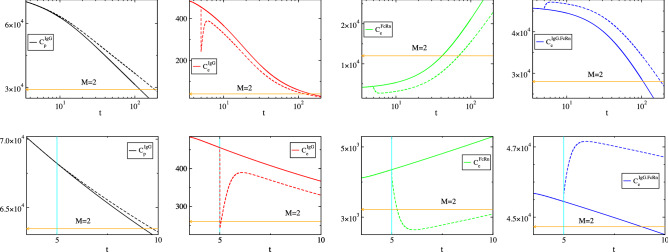
Concentration profiles for the reference case (parameter values in Table 1, solid curves) and for the case in which the reaction rate constant of $R^{3f}$ is doubled (dashed curves). The perturbation is enforced from $t=5\, \mathrm{h}$, as indicated by the vertical line. The indicated $M=1$ and $M=2$ periods refer to the reference case.

In order to validate these findings and explain the discrepancies between the reduced models in “Conventional reduced models” Section and the *Importance Index* findings in Fig. 6, the influence of reaction 1 and 3f in driving the system was investigated, by doubling their reaction rate constant from a specific point in time until the end of the process and by comparing the perturbed concentration profiles with the unperturbed ones. In general, a perturbation - such as the one considered here - will influence both the constraints and the manner in which the process evolves within these constraints. Naturally, the response of the constraints will manifest in accordance to the fast time scales that characterized their formation, while the response of the evolution within the constraints will be will manifest according to the slow time scales. The results such a comparison are shown in:

(i) Figure 7, where the effect of the perturbation imposed by doubling the rate constant of $R^1$, enforced from $t\,=\,0.1\,\mathrm{h}$ (where $M=1$) is displayed in the top row and that enforced from $t=5\, \mathrm{h}$ (where $M=2$) is displayed in the bottom row and
(ii) Figure 8, where the effect of the perturbation imposed by doubling the rate constant of $R^{3f}$, enforced from $t=5\, \mathrm{h}$ (where $M=2$) is displayed.

#### Perturbation on $R^1$

The top row of Fig. 7 shows that when the perturbation on $R^1$ is initiated at $t=0.1\, \mathrm{h}$ (where $M=1$), the perturbed profile of $C_e^{IgG}$ exhibits a sudden increase, while those of the other three variables exhibit a smooth departure, relative to the unperturbed profiles. The sudden increase of $C_e^{IgG}$ is due to the response to the perturbation of the equilibrium $R^1-(R^{3f}-R^{3b}) \approx 0$ in Eq. (37) that develops in the $M=1$ period:

45 $$\begin{aligned} \gamma k_1C^{IgG}_p - ( k_{on}C^{IgG}_e.C^{FcRn}_e-k_{off}C^{IgG.FcRn}_e)\approx 0 \end{aligned}$$

where $\gamma =1$ in the unperturbed case and $\gamma =2$ in the perturbed one. According to the $P^1_k$ values displayed in Fig. 5, the contribution of $R^1$ in this equilibrium is relatively small, but not negligible; i.e., at $t=0.1\, \mathrm{h}$ when the perturbation is enacted $P^1_1=0.15$, $P^1_{3f}=-0.50$ and $P^1_{3b}=0.35$. As a result, a perturbation on $R^1$ will produce a weak response. In addition, according to the $D^1_n$ values displayed in Fig. 5, the pointed variable for this equilibrium is $C_e^{IgG}$; $D^1_{C_e^{IgG}}\approx 1$. Therefore, it is this variable that will adjust the most as soon as the equilibrium in Eq. (45) is perturbed, exactly as displayed in the top row of Fig. (7); i.e., a sudden increase of $\gamma$ produces an equally fast increase of $C_e^{IgG}$, which is limited in size, given the contribution of $R^1$ to the equilibrium in Eq. (45). The displayed in Fig. 7 smooth increased rates of consumption of $C_p^{IgG}$ and $C_e^{FcRn}$ and of production of $C_e^{IgG.FcRn}$ and $C_e^{IgG}$ (the latter after the sudden increase due to the perturbed equilibrium), are all in agreement with the *Importance Index* findings reported in Fig. 6; i.e., $II^{IgG,e}_1<0$, $II^{FcRn,e}_1<0$, $II^{IgG.FcRn,e}_1>0$, $II^{IgG,e}_1>0$.

The bottom row of Fig. 7 shows that when the doubling of the reaction rate constant of $R^1$ is initiated at $t=5\, \mathrm{h}$ ($M=2$), the perturbed profile of $C_e^{IgG}$, $C_e^{FcRn}$ and $C_e^{IgG.FcRn}$ exhibit a sudden change and then a smooth variation, while the profile of $C_p^{IgG}$ exhibits a smooth departure, relative to the unperturbed profiles. The sudden changes are due to the the two equilibria $R^{3f}-R^{3b} \approx 0$ and $R^1-R^4+[c_2^2R^2+c^2_3(R^{3f}-R^{3b})] \approx 0$ in Eq. (44) that develop in the $M=2$ period:

46 $$\begin{aligned} k_{on}C^{IgG}_e.C^{FcRn}_e-k_{off}C^{IgG.FcRn}_e\approx 0 \qquad \quad \gamma k_1C^{IgG}_p - k_1C^{IgG.FcRn}_e +[\dots ] \approx 0 \end{aligned}$$

where $\gamma =1$ in the unperturbed case, $\gamma =2$ in the perturbed one and $[\dots ]$ denote smaller contributions. According to the $P^2_k$ values displayed in Fig. 5, the contribution of $R^1$ in the second equilibrium is significant; i.e., at $t=5\, \mathrm{h}$ when the perturbation is enacted $P^2_1=0.39$, $P^2_2=-0.13$, $P^2_{3f}=0.11$, $P^2_{3b}=-0.11$ and $P^2_4=-0.26$. In addition, according to the $D^2_n$ values displayed in Fig. 5 and Table 4, for the second equilibrium there two the pointed variables at the start of the $M=2$ period, $C_e^{FcRn}$ and $C_e^{IgG.FcRn}$, and only one during the remaining period, $C_e^{IgG.FcRn}$. Given that the perturbation is enacted right after the start of the $M=2$ period, a sudden increase of $\gamma$ will result in a sudden increase of $C_e^{IgG.FcRn}$, via the second equilibrium in Eq. (46) and a sudden decrease of $C_e^{FcRn}$ via the conservation law $C^{FcRn}_e+C^{IgG.FcRn}_e=FcRn_o$. In turn, this sudden increase of $C_e^{IgG.FcRn}$ will result to a sudden increase of $C_e^{IgG}$ via the first equilibrium in Eq. (46), given that this is the pointed variable, as shown in Fig. 5 and Table 4. The smooth increased rates of consumption of $C_p^{IgG}$, $C_e^{IgG}$ and $C_e^{IgG.FcRn}$ and production of $C_e^{FcRn}$, exhibited either right after the perturbation (applies to $C_p^{IgG}$) or right after the sudden response to the perturbation of the two equilibria (applies to $C_e^{IgG}$, $C_e^{IgG.FcRn}$ and $C_e^{FcRn}$) are in full agreement with the *Importance Index* findings reported in Fig. 6; i.e., $II^{IgG,p}_1<0$, $II^{IgG,e}_1<0$, $II^{IgG.FcRn,e}_1<0$ and $II^{FcRn,e}_1>0$.

#### Perturbation on $R^{3f}$

Figure 8 displays the response of the concentration profiles to the doubling of the reaction rate constant of $R^{3f}$, enacted at $t=5\, \mathrm{h}$ (where $M=2$). It is shown that the perturbation generates a sudden response of $C^{IgG}_e$, which is followed by a slower response of $C^{IgG}_e$, $C^{FcRn}_e$ and $C^{IgG.FcRn}_e$ and a subsequent even slower response of all four variables. These three successive types of responses are due to the fact that at the time when the perturbation is enacted there are two different equilibria established, which are characterized by two different time scales ($\tau _1\ll \tau _2$), and that the perturbation modifies both of them. In particular, in the $M=2$ period the established equilibria $R^{3f}-R^{3b} \approx 0$ and $R^1-R^4+[c_2^2R^2+c^2_3(R^{3f}-R^{3b})] \approx 0$, are expressed as:

47 $$\begin{aligned}&\gamma k_{on}C^{IgG}_e.C^{FcRn}_e-k_{off}C^{IgG.FcRn}_e\approx 0 \end{aligned}$$

48 $$\begin{aligned}&k_1C^{IgG}_p - k_1C^{IgG.FcRn}_e +[\dots + \gamma c^2_3 k_{on}C^{IgG}_e.C^{FcRn}_e +\dots ] \approx 0 \end{aligned}$$

so that $\gamma$ now associates to $R^{3f}$ ($\gamma =1$ in the unperturbed case, $\gamma =2$ in the perturbed one) and is present in the expression of both equilibria. According to the $P^1_k$ and $P^2_k$ values displayed in Fig. 5, the contribution of $R^{3f}$ in the first equilibrium in Eq. (47) is more significant than the second one in Eq. (48); i.e., at $t=5\, \mathrm{h}$ when the perturbation is enacted $P^1_{3f}=-0.49$ and $P^2_{3f}=0.11$. Therefore, the response to the perturbation considered here of the first equilibrium will be more pronounced than that of the second; both in the speed of its manifestation (since $\tau _1\ll \tau _2$) and in magnitude (since $|P^1_{3f}|\ll |P^2_{3f}|$). These features are displayed Fig. 8, where an increase of $\gamma$ is shown to generate initially a very fast decrease of $C^{IgG}_e$ (the variable exhibiting the largest $D^1_n$ values, according to Fig. 5) via the first equilibrium and a slower decrease of $C^{FcRn}_e$ and increase of $C^{IgG.FcRn}_e$ (the variables exhibiting the largest $D^2_n$ values, according to Fig. 5) via the second equilibrium and the conservation law $C^{FcRn}_e+C^{IgG.FcRn}_e=FcRn_o$. The evolution of the four variables after these two initial transients are in accordance to the *Importance Index* results in Fig. 6. Specifically, the consumption of $C^{IgG}_p$, $C^{IgG}_e$, $C^{IgG.FcRn}_p$ and the production of $C^{FcRn}_e$ slow down ($II_p^{IgG}>0$, $II_e^{IgG}>0$, $II_e^{IgG.FcRn}>0$ and $II_e^{FcRn}<0$).

### Discussion

The results presented in “The M = 1 period” to “The reactions driving the process” Sections demonstrated that CSP can algorithmically provide all information needed in order to acquire system-level understanding. CSP provided everything the cumbersome analytical methodologies might provide and many more. Specifically:

(i) The analytical methodology does not provide a method for the identification of the fast variables and fast reactions. These identifications are usually based on the experience and intuition of the investigator. In contrast, via the *Partitipation Index* $P^r_k$, the *Pointer* $D^n_i$ and *Time scale Participation Index* $J^n_k$, CSP can make these identifications algorithmically, as it was demonstrated in “The M = 1 period” and “The M = 2 period” Sections.
(ii) The analytic methodology assumes that a reaction deemed fast must be eliminated from the reduced model. As a result its influence on the slow evolution cannot be assessed. In contrast, CSP allows for a reaction to have a fast and a slow component, so its influence in shaping the constraints and in driving the system within these constraints can be assessed with the *Importance Index* $II^n_i$, as it was demonstrated in “[Sec Sec14]” Section.

The significance of the identifications in item (1) is profound, especially when the mathematical model of interest is large and complex. Item (2) addresses a feature that the conventional methodologies cannot handle; that is, the fact that reactions have usually a fast and a slow component, each of which might exercise a different influence. This feature was first encountered in the case of the glycolysis cycle, where the slow component of certain reactions were shown to promote a larger cycle, while their fast component were opposing this^39^. It was shown that an increase of the reaction rate constant of these reactions was promoting their fast component, reducing significantly the period of the cycle. Similar features are manifested in the simple PK model considered here, resulting to (1) the reversal of the influence of reactions 1 and 4 during the transition from the $M=1$ to the $M=2$ period and (2) the influence in the $M=2$ period of reaction 3, which was deemed equilibrated there; $R^{3f} \approx R^{3b}$.

Specifically, according to Fig. 6, towards the end of the $M=1$ period reaction 1 tends to decrease $C^{FcRn}_e$ and to increase $C^{IgG}_e$ and $C^{IgG.FcRn}_e$, while in the $M=2$ period this influence of reaction 1 reverses. A similar behavior is exhibited by reaction 4; i.e., in the $M=1$ period reaction 4 tends to increase $C^{FcRn}_e$ and to decrease $C^{IgG}_e$ and $C^{IgG.FcRn}_e$, while it tends to the opposite in the $M=2$ period. This feature is due to the fact that reactions 1 and 4 have a fast and a slow component, which tend to move the system in opposing directions. In particular, Fig. 6 shows that in the $M=1$ and $M=2$ periods reaction 1 contributes significantly to both $f^2$ and $f^3$, while has negligible contribution to the fastest amplitude $f^1$; as indicated by the large values of $P^2_1$ and $P^3_1$ and the negligible ones $P^1_1$. This implies that reaction 1 has non-negligible components only in the 2nd and 3rd CSP modes:

49 $$\begin{aligned} \mathbf {S}_1R^1\approx \mathbf {a}_2\left( \mathbf {b}^2 \cdot \mathbf {S}_1R^1\right) + \mathbf {a}_3\left( \mathbf {b}^3 \cdot \mathbf {S}_1R^1\right) \end{aligned}$$

where the first term in the RHS of this expression belongs to the relatively fast 2nd CSP mode $\mathbf {a}_2f^2$ ($f^2=\mathbf {b}^2 \cdot (\mathbf {S}_1R^1+\cdots )$) and the second term belongs to the relatively slow 3rd one $\mathbf {a}_3f^3$ ($f^3=\mathbf {b}^3 \cdot (\mathbf {S}_1R^1+\cdots )$). In the $M=1$ period, according to Eq. (29) the system is formally driven by both these CSP modes. However, according to the amplitude profiles in Fig. 4, $f^2\gg f^3$, so in this period it is mode $\mathbf {a}_2f^2$ that dominates. Therefore, the component of reaction 1 in the relatively fast 2nd mode exhibits much largest influence in the $M=1$ period, in comparison to its component in the relaatively slow 3rd mode. As a result, the value of the *Importance Index* $II^n_1$ in this period reflects the influence of the fast component of reaction 1. In the $M=2$ period the 2nd CSP mode is exhausted (i.e., $f^2\approx 0$) and the system is driven only by the 3rd mode $\mathbf {a}_3f^3$, in accordance to Eq. (30). As a result, the influence of reaction 1 is exercised only through its component in the relatively slow 3rd mode and $II^n_1$ there reflects the influence of the slow component of reaction 1. The fact that the $II^n_1$ values of $C^{IgG}_e$, $C^{FcRn}_e$ and $C^{IgG.FcRn}_e$ have opposite signs in the $M=1$ and $M=2$ periods, as displayed in Fig. 6, demonstrate that the fast and slow components of reaction 1 exhibit opposing influences. A similar explanation can be provided for the opposing influence of reaction 4 in the $M=1$ and $M=2$ periods, as shown in Fig. 6.

Reaction 3 exercises a significant influence in all three modes during the $M=2$ period, as it is demonstrated by the non-negligible *Amplitude Participation Indices* $P^n_{3f}$ and $P^n_{3b}$ that are displayed in Fig. 5 for $n=1,2,3$. This indicates that this reaction has non-negligible components in all three modes:

50 $$\begin{aligned} \mathbf {S}_{3}(R^{3f}-R^{3b}) =\mathbf {a}_1\left( \mathbf {b}^1 \cdot \mathbf {S}_3\left[ R^1-R^{3b}\right] \right) + \mathbf {a}_2\left( \mathbf {b}^2 \cdot \mathbf {S}_3\left[ R^1-R^{3b}\right] \right) + \mathbf {a}_3\left( \mathbf {b}^3 \cdot \mathbf {S}_3\left[ R^1-R^{3b}\right] \right) \end{aligned}$$

where $\mathbf {S}_{3}=\mathbf {S}_{3f}=-\mathbf {S}_{3b}$. The values of $P^1_{3f}\approx -0.5$ and $P^1_{3b}\approx 0.5$ in Fig. 5 for the fastest mode during the $M=2$ period indicate that this reaction is in partial equilibrium; $R^{3f} \approx R^{3b}$. However, this does not preclude this reaction having an influence in the slower modes, as it it demonstrated by the non-negligible *Importance Indices* $II^n_{3f}$ and $II^n_{3b}$, as shown in Fig. 6 and in more details in Fig. 8.

The traditional methods cannot distinguish the fast and slow components of the reactions. Therefore, the possibility that a change of the value of a parameter to have a certain influence in the short-run and the opposite in the long-run cannot be discovered. In contrast, the algorithmic CSP methodology can identify both (1) the component of a reaction that associates to a certain time scale via the *Partitipation Index* $P^r_k$, the *Pointer* $D^n_i$ and *Time scale Participation Index* $J^n_k$ and (2) the influence of this component in driving the process via the *Amplitude Participation Indices* $II^n_{k}$.

## Dominant dynamics in the M = 1 and M = 2 periods

It was demonstrated in the previous sections that CSP was able to algorithmically (1) identify the constraints that develop due to the fast dynamics and (2) recognize their nature; i.e., the fast variable, the fast reaction and the reactions participating in the constraints. It was also demonstrated that CSP can provide very accurate expressions for the constraints, which is reflected in the accuracy of the reduced model; see Eq. (31) for the general form of the constraints and the reduced model.

It will be demonstrated next that the CSP generated diagnostics, displayed in Figs. 2, 3, 4 and 5 and Tables 2 and 4, can provide the required knowledge in order to predict the response of the system when subjected to a change in the value of a parameter. The demonstration will address first the $M=1$ period and then the $M=2$ one.

### The M = 1 period

Let us first consider the $M=1$ period, for which is was shown that the process evolves within one constraint in the form of Eq. (35):

51 $$\begin{aligned} f^1= c^1_1R^1+c^1_2R^2+c^1_{3f}R^{3f}+c^1_{3b}R^{3b}+c^1_4R^4 \approx 0 \end{aligned}$$

**Figure 9:**
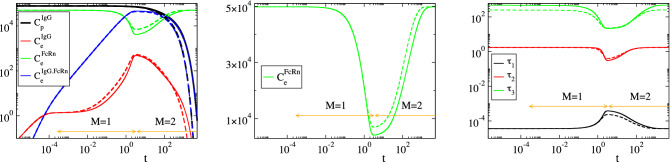
The reference (solid, $k_{off}$) and the perturbed (dashed, $2.0k_{off}$) concentration and time scale profiles. The indicated $M=1$ and $M=2$ periods refer to the reference case.

**Figure 10:**
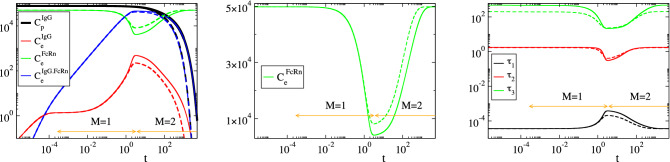
The reference (solid, $k_{deg}$) and the perturbed (dashed, $2.5k_{deg}$) concentration and time scale profiles. The indicated $M=1$ and $M=2$ periods refer to the reference case.

For this case, the Amplitude Participation Index $P^1_k$ displayed in Figure 5 shows that only reactions 1, 3f and 3b provide significant contributions in the occurring cancellations, i.e.:

52 $$\begin{aligned} \dfrac{c^1_1}{c^1_3} R^1+R^{3f}+R^{3b} = -k_1C^{IgG}_p+k_{on}C^{IgG}_e.C^{FcRn}_e-k_{off}C^{IgG.FcRn}_e \approx 0 \end{aligned}$$

where $c^1_{3f}=-c^1_{3b}=c^1_{3}$, since $\mathbf {S}_{3f}=-\mathbf {S}_{3b}$ and $c^i_k=\mathbf {b}^i\cdot \mathbf {S}_k$, and ${c^1_1}/{c^1_3}\approx -1$ throughout the $M=1$ period; see Table 2. As the Pointer $D^1_i$ diagnostics reported in Figure 5 show, the pointed variable for this equilibrium is $C^{IgG}_e$. Therefore it is this variable that will adjust the most in this period when a value of a parameter of those appearing in Eq. (52) is perturbed. For example, an increase of $k_{off}$ ($R^{3b}=k_{off}C^{IgG.FcRn}_e$) will cause an increase of $C^{IgG}_e$, while an increase of $k_{deg}$ ($R^2=k_{deg}C^{IgG}_e$) will have no effect, since it is not present in this equilibrium, Eq. (52). These conclusions are validated by the results displayed in Figs. 9 and 10, where concentration profiles computed with the reference parameter values in Table 1 are compared with profiles computed with perturbed values $2.0k_{off}$ and $2.5k_{deg}$). It is shown that variations of these two parameters have no other significant effect in the period where $M=1$, other than the increase of $C^{IgG}_e$ in the case of the perturbed value $2.0k_{off}$.

Regarding the influence of the perturbations to the time scales (1) $\tau _1$ that characterizes the development of the 1st equilibrium and (2) $\tau _2$ that characterizes the evolution of the system in the $M=1$ period, the changes in their profiles displayed in Figs. 9 and 10 are in full agreement with:

(i) The Time scale Participation Index $J^1_k$ diagnostics for $\tau _1$ reported in Fig. 5, according to which the related to the equilibrium in Eq. (52) time scale $\tau _1$ depends only on reaction 3f; i.e., is independent of $k_{off}$ or $k_{deg}$. Therefore, there will be no change in the $M=1$ period on how fast the first equilibrium will be established when $k_{off}$ or $k_{deg}$ are perturbed, as shown in Figs. 9 and 10.
(ii) The Time scale Participation Index $J^2_k$ diagnostics for $\tau _2$ reported in Fig. 5, according to which the time scale $\tau _2$, which characterizes the second mode that drives the system in the $M=1$ period (since $f^2\gg f^3$), depends mainly on reaction 4; i.e., is also independent of $k_{off}$ or $k_{deg}$. Therefore, there will be no change on how fast the system evolves in the $M=1$ period or on the time required for the second equilibrium to be established when $k_{off}$ or $k_{deg}$ are perturbed, as shown in Figs. 9 and 10.

### The M = 2 period

Let us next consider the $M=2$ period, for which is was shown that the process evolves within two constraints in the form of Eqs. (35) and (41):

53 $$\begin{aligned}&f^1= c^1_1R^1+c^1_2R^2+c^1_{3f}R^{3f}+c^1_{3b}R^{3b}+c^1_4R^4 \approx 0 \end{aligned}$$

54 $$\begin{aligned}&f^2= c^2_1R^1+c^2_2R^2+c^2_{3f}R^{3f}+c^2_{3b}R^{3b}+c^2_4R^4 \approx 0 \end{aligned}$$

The Amplitude Participation Index $P^1_k$ displayed in Fig. 5 shows that in the period considered here only reactions 3f and 3b provide significant contributions in the occurring cancellations in the first equilibrium ($R^{3f}-R^{3b}\approx 0$, Eq. (44)) and only reactions 1, 2 and 4 contribute to the second ($R^1 - R^4 +\left[ c_2^2R^2+\dots \right] \approx 0$, Eq. (44)), i.e.:

55 $$\begin{aligned}&\dfrac{c^1_{3f}}{c^1_{3b}}R^{3f}-R^{3b} = k_{on}C^{IgG}_e.C^{FcRn}_e-k_{off}C^{IgG.FcRn}_e \approx 0 \end{aligned}$$

56 $$\begin{aligned}&\dfrac{c^2_1}{c^2_4}R^1+\dfrac{c^2_2}{c^2_4}R^2+R^4 = -k_1C^{IgG}_p+k_1C^{IgG.FcRn}_e + \left[ ck_{deg}C^{IgG}_e+ \dots \right] \approx 0 \end{aligned}$$

where $c^1_{3f}=-c^1_{3b}$ and ${c^2_1}/{c^2_4}\approx -1$ throughout the $M=2$ period and $c=c^2_2/c^2_4>0$; see Table 4. As the Pointers $D^1_i$ and $D^2_i$ reported in Fig. 5 indicate, the pointed variable for the 1st equilibrium throughout the period is $C^{IgG}_e$ and for the 2nd equilibrium is mainly $C^{IgG.FcRn}_e$ and at the start of the period $C^{FcRn}_e$ as well. Therefore, these are the variables that will adjust the most in this period when a value of a parameter is perturbed.

Considering the increase of $k_{off}$ ($R^{3b}=k_{off}C^{IgG.FcRn}_e$), Eq. (55) shows that this will cause an increase of the pointed variable $C^{IgG}_e$ via the 1st equilibrium. This increase will cause a decrease of $C^{IgG.FcRn}_e$ via the 2nd equilibrium Eq. (56), which in turn will cause an increase of $C^{FcRn}_e$ via the conservation law $C^{FcRn}_e+C^{IgG.FcRn}_e=FcRn_o$. These CSP-based predictions are in full agreement with the findings displayed in Figure 9.

Considering now the increase of $k_{deg}$ ($R^2=k_{deg}C^{IgG}_e$), Eq. (56) shows that this will cause a decrease of the pointed variable $C^{IgG.FcRn}_e$ via the 2nd equilibrium, which in turn will cause an increase of $C^{FcRn}_e$ via the conservation law $C^{FcRn}_e+C^{IgG.FcRn}_e=FcRn_o$. The decrease of $C^{IgG.FcRn}_e$ and the increase of $C^{FcRn}_e$ will in turn cause a decrease of the pointed variable $C^{IgG}_e$ via the 1st equilibrium Eq. (55). Again, these CSP-based predictions are in full agreement with the findings displayed in Figure 10.

Note that in both perturbations considered, the $C^{IgG}_p$ profile follows that of $C^{IgG.FcRn}_e$. This feature is in accordance to the 2nd equilibrium Eq. (56), in which the contribution of reaction 2 decreases as the process moves deeper into the $M=2$ period; i.e., $P^2_2 \rightarrow 0$, as shown in Fig. 5.

Regarding the influence of the perturbations to the time scales (1) $\tau _1$ and $\tau _2$ that characterize the development of the 1st and 2nd equilibria and (2) $\tau _3$ that characterizes the evolution of the system in the $M=2$ period, the changes in their profiles displayed in Figs. 9 and 10 are in full agreement with:

(i) The Time scale Participation Indices $J^1_k$ and $J^2_k$ diagnostics reported in Figure 5, according to which the related to the first equilibrium time scale $\tau _1$ depends only on reaction 3f and the related to the second equilibrium time scale $\tau _2$ depends basically on reaction 4 (i.e., both are independent of $k_{off}$ or $k_{deg}$).
(ii) The Time scale Participation Index $J^3_k$ diagnostics reported in Figure 5, according to which the time scale $\tau _3$ that characterizes the only active mode depends mainly on all reactions; i.e., it depends on $k_{off}$ and $k_{deg}$. Since $J^3_{3b}$ and $J^3_2$ are both negative, increasing $k_{off}$ or $k_{deg}$ ($R^{3b}=k_{off}D$, $R^2=k_{deg}B$) will result in a faster $\tau _3$; i.e., the process will evolve faster in the $M=2$ period.

These predictions are validated by the results shown in Figs. 9 and 10. In particular, it is shown in these figures the fastest consumption of $C^{IgG}_p$, $C^{IgG}_e$ and $C^{FcRn}_e$ and the fastest reach to steady state of $C^{FcRn}_e$ in the $M=2$ period, which are due to the faster characteristic time scale $\tau _3$.

## Conclusions

A demonstration is provided here on how algotithmic multi-scale analysis can provide system-level understanding. This work refers to the traditional analytic and to an algorithmic (CSP) treatment of a simple model that exhibits multi-scale dynamics. It is shown that CSP provides algorithmically all the results that are provided by analytical techniques along with additional ones, that cannot be obtained analytically. In contrast to the traditional analytical treatment, CSP was shown to require no input or experience from the investigator.

In a multi-scale process, the fast and slow dynamics exhibit distinct influence: (1) the fast dynamics generates constraints in which the process evolves and (2) this evolution is governed by a slow model (free of the fast dynamics). A perturbation along the fast components of the model (reactions) will have an immediate effect that will affect the fast variables, since it will modify the existing constraints, the development of which is characterized by the fast time scales. In contrast, a perturbation along the slow components will have a gradual effect and can potentially affect all variables, since it will modify the evolution of the process within the established constraints. A multi-scale analysis aims in identifying (1) the constraints, (2) the slow system that governs the evolution within the constraints, (3) the reactions whose fast component is responsible for the development of the constraints, (4) the reactions whose slow component determines the slow evolution, (5) the reactions that can be ignored; i.e., those that do not contribute to the fast or slow dynamics and (6) the fast variables; i.e., those that are “slaved” to the rest. CSP provides all these in an algorithmic fashion, so no input from the investigator is required and the analysis can be repeated for any parameter set of interest. The conclusions reached for a given set of parameters are valid for in a large domain of the parameter space, in which basic dynamics properties (such as time scale separation) are not altered.

It was demonstrated that the CSP diagnostics provide all necessary information to predict the response of the system when a change in the parameters is introduced with the use of specific algorithmic tools; $P^n_k$ (identifies the reactions participating in the n-th equilibrium), $D^n_i$ (identifies the variables that will adjust the most when the system is perturbed), $J^n_k$ (identifies the reactions that generate the n-th time scale) and $II^n_k$ (identifies the reactions that drive the n-th variable within the established equilibria). These numerical diagnostics can provide all required information in order to acquire system-level understanding and seek ways to redesign the process.

Finally, it was shown that CSP can distinguish the dual role a reaction might exhibit, via its opposing influence in the fast and slow dynamics. This is a feature that popular methodologies (such as, paper-and-pencil singular perturbation analysis, QSSA and PEA) cannot handle, since they cannot consider reactions having a significant fast and slow component. The same applies to sensitivity analysis, which cannot distinguish fast and slow reactions.

It is important to note that our automatic algorithmic analysis came to some conclusions that are very similar to the ones a pharmacokineticist would have derived based on detailed manual analysis of the system. For example, in the very early time points after antibody administration (i.e., $M=0$ period) the entry of antibody from plasma to endosome via pinocytosis (i.e., reaction 1) affects plasma PK the most, and minimizing this process with help achieve higher plasma concentrations. This aspect can be leveraged to design better antibodies for more efficient elimination of pathogenic targets. For example, recently sweeping antibodies have been developed that utilize surface FcRn or Fc-gamma receptors to increase cellular uptake of antibodies and associated pathogenic protein molecules (e.g., PCSK9, C5 etc.)^61,62^. Literature and our lab’s recent data, which show that positively charged antibody molecules demonstrate a higher cellular uptake rate and a reduced plasma exposure, also support the conclusion derived by the model over here^63,64^. As the time progresses (i.e., $M=1$ period), reducing the pinocytosis in the cells and increasing the recycling of FcRn bound mAbs (i.e., strengthening reaction 4) both will help maintain higher plasma exposure of mAbs. Even at later time points (i.e., $M=2$ period), weakening reaction 1, strengthening reaction 4, weakening reaction 2 (i.e., reducing lysosomal degradation rate of mAbs), and strengthening reaction 3f along with weakening reaction 3b, will all help improve the plasma exposure of mAbs. This aspect of the process has been heavily utilized by the protein engineers to develop antibodies with very long half-lives. For example, mutations (e.g., YTE, LS etc.)^46,65^ have been introduced in the Fc region of the antibodies to improve their binding to the FcRn at pH 6.0, leading to half-life of several months (compared to 3 weeks for wild-type antibodies in human). Our analysis also provides additional insight not generated by other methods. For example, identification of the period in which the influence of each reaction in increasing plasma PK of mAb is exercised (e.g., reaction 4 becomes influential when enough FcRn bound mAb complex has been accumulated). These unprecedented insights can further stimulate protein engineering efforts to discover and develop better antibody-based drug molecules.

This work demonstrates the ability of CSP in fully analyzing complex Pharmacokinetic and Quantitative Systems Pharmacology models. Due to the algorithmic nature of CSP, the type of analysis introduced here can be extended to much more complex models; i.e., models that include target mediated drug disposition (TMDD) to characterize the PK of target binding antibodies and antibody drug conjugates (ADC)^66–69^. The new features introduced by these models will be represented as additional terms in the governing Eq. (1). The influence of these terms can be easily assessed by the algorithmic tools of CSP.

## Supplementary Information

Supplementary Information.

## Data Availability

The datasets generated during and/or analysed during the current study are available in the CSP_PK_IgG repository, https://github.com/patsatzisdim/CSP_PK_IgG.
