# Supplementary material for: Algorithmic multiscale analysis for the FcRn mediated regulation of antibody PK in human
Source: Sci Rep. 2022 Apr 13;12:6208. doi: 10.1038/s41598-022-09846-x (PMC9008124; doi:10.1038/s41598-022-09846-x)
Supplement: Supplementary file 1 — Supplementary Information. [file 41598_2022_9846_MOESM1_ESM.pdf]

# Supplement

The CSP vectors $\mathbf{a}_i$ and $\mathbf{b}^i$ ($i = 1, \ldots, N$) can be approximated to leading order accuracy by the right and left, respectively, eigenvectors of the $N \times N$-dim. Jacobian $\mathbf{J}$ of $\mathbf{g}$; i.e., $\mathbf{a}_i = \boldsymbol{\alpha}_i$ and $\mathbf{b}^i = \boldsymbol{\beta}^i$ [1, 2, 3].

Assuming there are $K$ reactions in the mechanism, the equilibria among the reactions that are established by the fast dynamics are expressed by the $M$ constraints in Eq. (31) $f^r = c_1^r R^1 + \ldots + c_K^r R^K \approx 0$ ($r = 1, \ldots, M$ and $c_k^r = \boldsymbol{\beta}^r \cdot \mathbf{S}_k$). The reactions that contribute significantly to the formation of each of the $M$ constraints are identified by the *Amplitude Participation Index* (*API*):

$$P_k^r = \frac{(\boldsymbol{\beta}^r \cdot \mathbf{S}_k) R^k}{\sum_{i=1}^{K} |(\boldsymbol{\beta}^n \cdot \mathbf{S}_i) R^i|} \qquad (k = 1, \ldots, K) \tag{1}$$

where by definition $\sum_{k=1}^{K} |P_k^r| = 1$ [4, 5, 6]. The relative contribution of the $k$-th reaction to the cancellations among the additive terms in $f^r \approx 0$ is thus measured by $P_k^r$, which can be either positive or negative; the sum of positive and negative terms equaling $\pm 0.5$, respectively.

The formation of the $M$ constraints is characterized by the $M$ fastest timescales, while the dynamics of the slow system in Eq. (31) by the fastest of the $N - M$ slow ones. The timescales are approximated by the inverse of the eigenvalues of the Jacobian $\mathbf{J}$, $\tau_n = |\lambda_n|^{-1}$ ($n = 1, \ldots, N$). The CSP diagnostic tool, *Time scale Participation Index* (*TPI*), identifies the reactions significantly contributing to the generation of the timescales:

$$J_k^n = \frac{h_k^n}{\sum_{i=1}^{K} |h_i^n|} \qquad (k = 1, \ldots, K) \tag{2}$$

where $\lambda_n = h_1^n + \ldots + h_K^n$ and by definition $\sum_{k=1}^{K} |J_k^n| = 1$ [7, 5, 8]. $h_k^n$ denotes the contribution of the $k$-th reaction to the $n$-th eigenvalue and can be calculated as $h_k^n = \boldsymbol{\beta}^n \nabla \left(\mathbf{S}_k R^k\right) \boldsymbol{\alpha}_n$, where $\sum_{k=1}^{K} \nabla \left(\mathbf{S}_k R^k\right)$ provides the Jacobian $\mathbf{J}$ of $\mathbf{g}$. $h_k^n$ can be either positive or negative and therefore, a negative (positive) $J_k^n$ implies that the $k$-th reaction contributes to a dissipative (explosive) character of the $n$-th timescale $\tau_n$. By definition, dissipative (explosive) timescales relate to the components of the system that tend to drive it towards (away from) equilibrium [2, 4].

Each CSP mode is associated differently to each metabolic species; e.g., a CSP mode can be related mostly to the $i$-th variables and much less to the rest. The relation of the $n$-th CSP mode $\mathbf{a}_n f^n$ to the various variables is identified by the *Pointer* (*Po*):

$$\mathbf{D}^n = diag\left[\boldsymbol{\alpha}_n \boldsymbol{\beta}^n\right] = \left[\alpha_n^1 \beta_1^n, \alpha_n^2 \beta_2^n, \ldots, \alpha_n^N \beta_N^n\right] \tag{3}$$

where, due to the orthogonality condition $\boldsymbol{\beta}^i \cdot \boldsymbol{\alpha}_j = \delta_j^i$, the sum of all $N$ elements of $\mathbf{D}^n$ equals unity, i.e. $\sum_{i=1}^{N} \alpha_n^i \beta_i^n = 1$ [9, 4, 6, 10]. A relatively large value of $\alpha_n^i \beta_i^n$ indicates that the $i$-th species is strongly associated to $n$-th CSP mode and the $n$-th timescale. A value of $D_i^n$ close to unity suggests the validity of the *Quasi Steady-State Approximation* (QSSA) for the $i$-th variable, while only two large values of $D_i^n$ indicate the validity of the *Partial Equilibrium Approximation* (PEA) [10]. The variables that are pointed by the *Pointer* by the $M$ exhausted

modes are those that are "slaved" to the remaining variables via the equilibrium relations $f^r \approx 0$ in Eq. (31).

When the $M$ constraints are established, the system evolves along them according to the differential equation in Eq. (31). However, each reaction contributes to a different degree to each metabolic species. The contribution of the reactions to the slow evolution of a particular metabolic species is identified by the *slow Importance Index* (*II*):

$$I_k^n = \frac{\sum_{s=M+1}^{N} \alpha_s^n (\boldsymbol{\beta}^s \cdot \mathbf{S}_k) R^k}{\sum_{j=1}^{K} | \sum_{s=M+1}^{N} \alpha_s^n (\boldsymbol{\beta}^s \cdot \mathbf{S}_j) R^j |} \qquad (n = 1, \ldots, N, \quad k = 1, \ldots, K) \tag{4}$$

where by definition, $\sum_{k=1}^{K} |I_k^i| = 1$ [4, 5]. $I_k^n$ provides a measure of the relative importance of the $k$-th reaction to the production (when positive) or consumption (when negative) of the $n$-th metabolic species [9, 4, 6].

The identification of the number $M$ of the exhausted modes is algorithmically provided by CSP through the criterion:

$$\left| \tau_{M+1} \sum_{i=1}^{M} \boldsymbol{\alpha}_i f^i \right| < e_{rel} \boldsymbol{y} + \boldsymbol{e}_{abs}, \tag{5}$$

where $e_{rel}$ and $\boldsymbol{e}_{abs}$ denote relative and absolute errors, respectively. Given the solution of the system at a specific time point, the criterion in Eq. (5) identifies the $M$ fast timescales, according to the desired accuracy.

# Bibliography


[1] S. Lam, D. Coussis, Conventional asymptotics and computational singular perturbation for simplified kinetics modelling, in: Reduced kinetic mechanisms and asymptotic approximations for methane-air flames, Springer, 1991, pp. 227–242.

[2] S. H. Lam, D. A. Goussis, Understanding complex chemical kinetics with computational singular perturbation, Symposium (International) on Combustion 22 (1) (1989) 931–941.

[3] E. A. Tingas, D. C. Kyritsis, D. A. Goussis, Ignition delay control of DME/air and EtOH/air homogeneous autoignition with the use of various additives, Fuel 169 (2016) 15–24.

[4] S. H. Lam, D. A. Goussis, The CSP method for simplifying kinetics, International Journal of Chemical Kinetics 26 (4) (1994) 461–486.

[5] D. A. Goussis, H. N. Najm, Model reduction and physical understanding of slowly oscillating processes: the circadian cycle, Multiscale Modeling & Simulation 5 (4) (2006) 1297–1332.

[6] M. Valorani, H. N. Najm, D. A. Goussis, CSP analysis of a transient flame-vortex interaction: time scales and manifolds, Combustion and Flame 134 (1-2) (2003) 35–53.

[7] D. Goussis, G. Skevis, Nitrogen chemistry controlling steps in methane-air premixed flames, Computational fluid and solid mechanics 1 (2005) 650–653.

[8] D. J. Diamantis, E. Mastorakos, D. A. Goussis, H2/air autoignition: the nature and interaction of the developing explosive modes, Combustion Theory and Modelling 19 (3) (2015) 382–433.

[9] D. Goussis, S. Lam, A study of homogeneous methanol oxidation kinetics using CSP, Symposium (International) on Combustion 24 (1) (1992) 113–120.

[10] D. A. Goussis, Quasi steady state and partial equilibrium approximations: their relation and their validity, Combustion Theory and Modelling 16 (5) (2012) 869–926.
